# Notch sensitivity of orthotropic solids: interaction of tensile and shear damage zones

**DOI:** 10.1007/s10704-018-0296-5

**Published:** 2018-07-05

**Authors:** Harika C. Tankasala, Vikram S. Deshpande, Norman A. Fleck

**Affiliations:** Engineering Department, Cambridge University, Trumpington St., Cambridge, CB2 1PZ UK

**Keywords:** Notch sensitivity, Orthotropy, Crack extension, Splitting, Cohesive zone

## Abstract

The macroscopic tensile strength of a panel containing a centre-crack or a centre-hole is predicted, assuming the simultaneous activation of multiple cohesive zones. The panel is made from an orthotropic elastic solid, and the stress raiser has both a tensile cohesive zone ahead of its tip, and shear cohesive zones in an orthogonal direction in order to represent two simultaneous damage mechanisms. These cohesive zones allow for two modes of fracture: (i) crack extension by penetration, and (ii) splitting in an orthogonal direction. The sensitivity of macroscopic tensile strength and failure mode to the degree of orthotropy is explored. The role of notch acuity and notch size are assessed by comparing the response of the pre-crack to that of the circular hole. This study reveals the role of the relative strength and relative toughness of competing damage modes in dictating the macroscopic strength of a notched panel made from an orthotropic elastic solid. Universal failure mechanism maps are constructed for the pre-crack and hole for a wide range of material orthotropies. The maps are useful for predicting whether failure is by penetration or kinking. Case studies are developed to compare the predictions with observations taken from the literature for selected orthotropic solids. It is found that synergistic strengthening occurs: when failure is by crack penetration ahead of the stress raiser, the presence of shear plastic zones leads to an enhancement of macroscopic strength. In contrast, when failure is by crack kinking, the presence of a tensile plastic zone ahead of the stress raiser has only a mild effect upon the macroscopic strength.

## Introduction

Macroscopic notches in a panel induce stress concentrations and reduce its load-carrying capacity. The strength reduction depends upon the degree of material non-linearity and on the failure path. Recall that a circular hole in an infinite elastic sheet has an elastic stress concentration factor of 3 (see for example, Peterson 1974) and this leads to a knock-down factor of 3 in strength, assuming a point-wise, stress-based failure criterion. In contrast, the macroscopic tensile strength of an infinite sheet, made from a rigid, ideally plastic solid, is not reduced by the introduction of a circular hole. Engineering composites fail in a more complex manner with the simultaneous activation of a number of damage mechanisms such as fibre pull-out and splitting along the interface between fibre and matrix. These failure processes can be idealised by tensile and shear cohesive zones, for example.

**Fig. 1:**
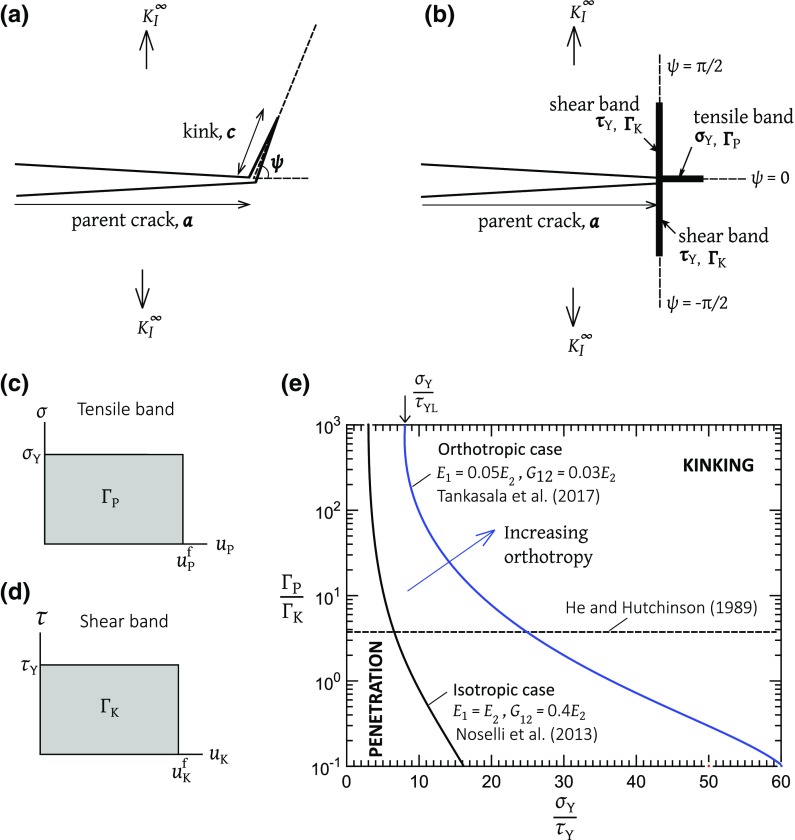
Crack kinking in solids. **a** General case as considered by He and Hutchinson (1989); **b** tensile band along $\psi =0$ and shear bands along $\psi = \pm \pi /2$, as considered by Tankasala et al. (2017); cohesive zone laws for **c** tensile band and **d** shear band; **e** the boundary between penetration and kinking at the tip of a semi-infinite mode I crack

Consider the prototypical problem of crack kinking from the tip of a parent crack of length *a*, as shown in Fig. 1a. The parent crack is subjected to macroscopic mode I loading, as dictated by the stress intensity factor $K_{I}^{\infty }$. A traction-free kink of length *c* is placed at the the tip of the parent crack, at an orientation $\psi$ to the main cracking plane. For a homogeneous isotropic solid, He and Hutchinson (1989) obtained expressions for the energy release rate at the tip of the parent crack, $G^\mathrm{tip}(\psi )$, and assumed that the direction of kinking is dictated by the orientation $\psi$ that maximizes $G^\mathrm{tip}$, such that $\partial G^\mathrm{tip}/\partial \psi =0$; see Fig. 1a. Alternatively, consider a solid with a directional toughness such that the toughness $\mathrm {\Gamma }_\mathrm{P}$ along the penetration direction $\psi =0$ differs from the toughness $\mathrm {\Gamma }_{\mathrm{K}}$ along the orthogonal kinking direction $\psi =\pi /2$. The solid may be isotropic or anisotropic in elastic response. He and Hutchinson (1989) assume that penetration occurs when

1 $$\begin{aligned} \dfrac{G^\mathrm{tip}(\psi =0)}{G^\mathrm{tip}(\psi =\pi /2)} > \dfrac{\mathrm {\Gamma }_{\mathrm{P}}}{\mathrm {\Gamma }_{\mathrm{K}}} \end{aligned}$$

whereas kinking occurs when

2 $$\begin{aligned} \dfrac{G^\mathrm{tip}(\psi =0)}{G^\mathrm{tip}(\psi =\pi /2)} < \dfrac{\mathrm {\Gamma }_{\mathrm{P}}}{\mathrm {\Gamma }_{\mathrm{K}}} \end{aligned}$$

We emphasize that this purely energetic approach is consistent with the physical interpretation that a single flaw of orientation $\psi =0$ exists along the crack front, and a competing flaw of orientation $\psi =\pi /2$ exists elsewhere along the crack front. The subsequent crack path is either along $\psi =0$ or $\psi =\pi /2$. For an isotropic solid, penetration along $\psi =0$ occurs for ${\mathrm {\Gamma }_{\mathrm{P}}}/{\mathrm {\Gamma }_{\mathrm{K}}}$ $<3.8$. This energetic approach is appropriate for brittle monolithic ceramics or for interfacial fracture along the brittle interface between two ceramics. A systematic methodology has emerged for a whole class of problems, as detailed in the recent monograph by Begley and Hutchinson (2017). In contrast, a large class of engineering solids fail by the *simultaneous* development of one or more inelastic damage zones at the crack tip.

Consider for example, the case of unidirectional long fibre composites with a ceramic or polymeric matrix. Typically, these composites are stiff and strong along the fibre direction in comparison to the transverse direction. Natural woods also fall into this class. Depending on the notch geometry and material properties of the constituents (matrix, reinforcements, and interfaces), damage at the notch tip can occur by: (i) matrix cracking, (ii) splitting along the fibre direction, (iii) cracking transverse to fibre direction (Zok 2006; Ashby et al. 1985), (iv) fibre pull-out or microbuckling, or (v) interface cracking and/or debonding (Fleck and Zhao 2000; Soboyejo 2002; Heredia et al. 1994; Green et al. 2007; Nambu et al. 2009; Chan et al. 1993; Hwu and Derby 1999; Cao and Evans 1991). All these damage phenomenon can occur simulateously. The generation of shear bands along the fibre direction has been documented recently for carbon-fibre reinforced polymer (CFRP) composites by Tan et al. (2015) and Cox and Yang (2006), inter alia; this damage mode emerges from the edge of a hole or from the tip of a pre-crack, and may accompany tensile rupture, as documented in detail by Tan et al. (2017) and in the review by Zok (2006). The interaction of these coexisting damage modes deserves consideration in the modelling of composite failure. The purpose of the present study is to explore the significance of *simultaneous damage* along two fracture paths from a stress-raiser such as a sharp crack or a circular hole under remote tension. The failure mechanisms are lumped into two broad categories: (i) tensile damage ahead of the notch and (ii) shear damage orthogonal to the notch but originating at the notch tip.

The transition of notch behaviour from brittle to ductile for fibre-reinforced ceramic-matrix composites has been addressed in part by Suo et al. (1993), He et al. (1994), Connell et al. (1994) and McNulty et al. (1999). Assume that the composite fails by the development of a tensile inelastic zone associated with cracking of the brittle matrix and crack-bridging by fibre pull-out. This damage region is commonly modelled by a traction versus separation law for an equivalent tensile cohesive zone. Write $\sigma _{\mathrm{o}}$ as the maximum traction in the cohesive zone, $u_{\mathrm{f}}$ as a critical opening of the cohesive zone, and *E* as Young’s modulus of the composite, assumed isotropic. Then, dimensional analysis demands that the macroscopic net-section tensile strength $\sigma _\mathrm{max}^{\infty }$ in the presence of a notch (hole or crack) of characteristic length *a*, scales as

3 $$\begin{aligned} \dfrac{\sigma _{\text {max}}^{\infty }}{\sigma _{\mathrm{o}}} = f\left( \dfrac{a\sigma _{\mathrm{o}}}{Eu_{\mathrm{f}}} \right) \end{aligned}$$

with the function *f* taking the limiting values of $f=1$ when $a\sigma _{\mathrm{o}}/Eu_{\mathrm{f}}=0$, and $f=0$ when $a\sigma _\mathrm{o}/Eu_{\mathrm{f}}=\small \infty$. The significance of the notch size *a* in relation to a material length scale $Eu_{\mathrm{f}}/\sigma _{\mathrm{o}}$ was first recognized by Cottrell (1963) and now underpins design codes such as the structural integrity assessment procedure R6 (2001) for metallic structures.

The present study builds upon the recent study of Noselli et al. (2013) wherein the competition between crack kinking along a shear damage zone versus penetration along a tensile damage zone was studied for a semi-infinite parent crack in an isotropic solid under mode I loading. This was extended by Tankasala et al. (2017) to include the role of elastic orthotropy on the competition between the damage modes. The two damage zones were idealized by cohesive zones, each of finite strength and finite toughness, and the competition of penetration versus kinking was determined as a function of the relative strength and relative toughness of the cohesive zones. Synergistic toughening was observed, whereby the parent crack is shielded by the activation of both tensile and shear cohesive zones, and the macroscopic toughness is elevated. In the current study, we extend the work of Tankasala et al. (2017) to explore the role of elastic orthotropy on crack path selection when an inelastic penetration band and an inelastic kink band coexist at the tip of a finite crack, or at the edge of a circular hole. The cohesive zone model of Tankasala et al. (2017) for a semi-infinite mode I parent crack is now reviewed.

### Cohesive zone model for simultaneous damage at the tip of a long crack in an orthotropic solid

Consider a mode I crack in an orthotropic elastic solid, with a tensile band directly ahead of the crack tip and two shear bands orthogonal to the cracking plane, as shown in Fig. 1b. In response to remote mode I loading, the **tensile band** behaves as a bridged mode I crack. The shear traction on the tensile band vanishes due to symmetry in both geometry and loading. The tensile band is idealized as rigid, perfectly plastic and of strength $\sigma _{\mathrm{Y}}$ for any finite opening $u_{\mathrm{P}}$ less than a critical value $u^\mathrm{f}_{\mathrm{P}}$, as illustrated in Fig. 1c. Failure of the cohesive zone is triggered by the condition $u_{\mathrm{P}}=u^\mathrm{f}_{\mathrm{P}}$ at the physical crack tip. The work of fracture for unit extension of crack along the tensile band is $\mathrm {\Gamma }_{\mathrm{P}} = \sigma _{\mathrm{Y}}u_{\mathrm{P}}^\mathrm{f}$. Likewise, each **shear band** is assumed to behave as a bridged mode II crack. It has a strength $\tau _{\mathrm{Y}}$ for a sliding displacement $u_{\mathrm{K}}$ less than the critical value $u^\mathrm{f}_{\mathrm{K}}$, see Fig. 1d. The shear cohesive zone fails when $u_{\mathrm{K}}=u^\mathrm{f}_{\mathrm{K}}$ at the crack tip, and gives rise to a finite toughness $\mathrm {\Gamma }_{\mathrm{K}}= \tau _{\mathrm{Y}}u_{\mathrm{K}}^\mathrm{f}$.

Recall that the purely energetic criterion Eq. (1), as proposed by He and Hutchinson (1989), gives the critical interface toughness ratio ${\mathrm {\Gamma }_{\mathrm{P}}}/{\mathrm {\Gamma }_{\mathrm{K}}}$ $=3.8$ for crack kinking in an isotropic solid. In contrast, the cohesive zone approach of Noselli et al. (2013) predicts that the active mode of failure between penetration and kinking in an isotropic solid depends upon *both* the interface toughness ratio ${\mathrm {\Gamma }_{\mathrm{P}}}/{\mathrm {\Gamma }_{\mathrm{K}}}$ and the interface strength ratio ${\sigma _{\mathrm{Y}}}/{\tau _{\mathrm{Y}}}$ in the manner as plotted in Fig. 1e. Now consider the case of an orthotropic elastic solid, with the parent crack aligned with the minor principal direction of the material $x_{1}$. Tankasala et al. (2017) considered the case with Young’s moduli $E_{1}=0.05E_{2}$ and shear modulus $G_{12}=0.03E_{2}$ and found that the regime of kinking shrinks in comparison to the isotropic case, see Fig. 1e. In the current study, we proceed to consider crack path selection for a finite pre-crack or a circular hole in an orthotropic solid.

### Scope of study

**Fig. 2:**
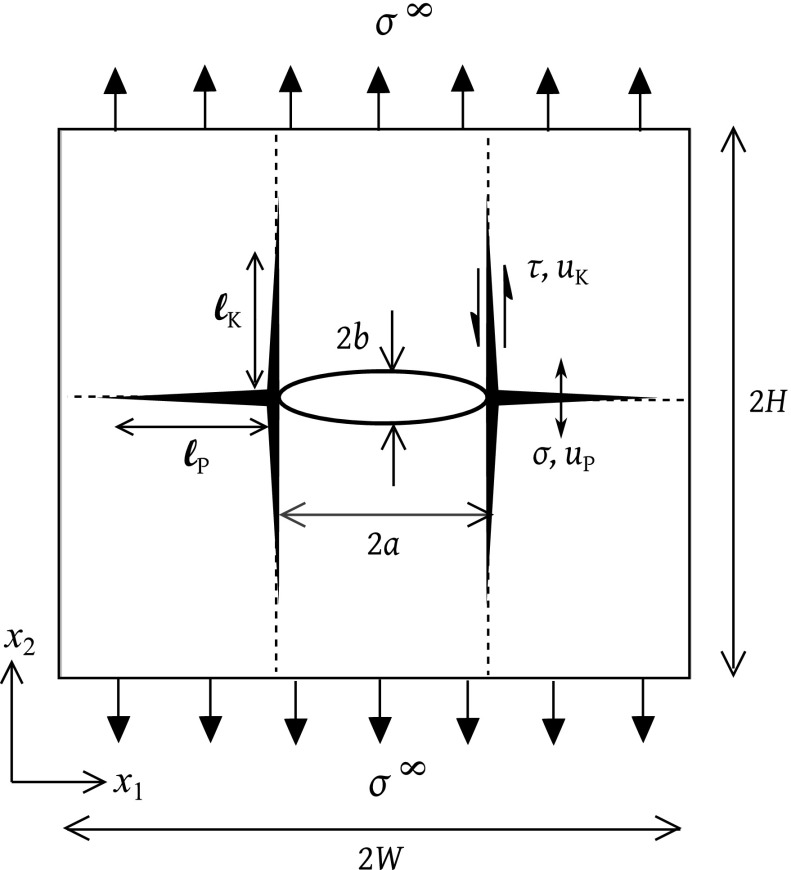
Centre-notched panel subjected to uniform tension

The interplay between a tensile zone ahead of the tip of an elliptical notch and a shear zone orthogonal to the plane of the notch is explored for the case of remote tensile loading of a panel, see Fig. 2. The general case is that of an elliptical notch of major axis 2*a* and minor axis 2*b*. Two notch geometries are considered in detail in the present study: a sharp crack ($b/a =0$) and a circular hole ($b/a=$1). Throughout this study, we shall assume that the minor axis of the notch is aligned with the loading direction $x_{2}$ which is also the major principal direction of the solid.^1^ The panel is of finite width 2*W* and height 2*H* such that $a/W=a/H=0.05$. This choice is made to avoid (where possible) the finite size of the specimen in relation to the initial notch length. Tensile damage ahead of the notch-tip, and shear damage on an orthogonal plane originating at the notch-tip, are represented by two ductile interfaces, as shown in Fig. 2. The nature of the cohesive zone law is sensitive to the failure mechanism exhibited by the material system. We restrict attention to the case when the failure mechanism of the kink bands is shear dominated i.e., the kinks grow with negligible opening.

Failure from the notch tip can develop in two ways: (i) a penetration mode of failure (when the tensile zone fails first) and (ii) a kinking mode of failure (when the shear zone fails first). The dominant mode depends upon (i) the relative strength and toughness of the competing zones (see for example Tankasala et al. 2017; Parmigiani and Thouless 2006), (ii) the nature of the stress raiser, i.e., crack or hole, and (iii) the degree of material orthotropy. All three aspects are considered herein.

## Micromechanical model

Figure 2 defines the problem. It is assumed that a tensile damage zone exists ahead of the notch while shear bands exist orthogonal to the notch plane, as shown in Fig. 2. As described earlier in Sect. 1.1, the tensile band behaves as a bridged mode I crack of tensile strength $\sigma _{\mathrm{Y}}$ and mode I toughness $\mathrm {\Gamma }_{\mathrm{P}} = \sigma _{\mathrm{Y}}u^\mathrm{f}_{\mathrm{P}}$ where $u^\mathrm{f}_{\mathrm{P}}$ is the critical opening displacement at the root of the notch. Likewise, the shear band is a bridged mode II crack of shear strength $\tau _{\mathrm{Y}}$ and mode II toughness $\mathrm {\Gamma }_{\mathrm{K}}= \tau _{\mathrm{Y}}u^\mathrm{f}_{\mathrm{K}}$. Here, $u^\mathrm{f}_{\mathrm{K}}$ is the critical sliding displacement at the root of the notch. The assumption that the inclined kink bands are shear bands follows the analysis of Chan et al. (1993), and is expected to be appropriate for ductile kink band behaviour (such as in MMCs). Other choices of cohesive zone have been considered in the literature, see for example Parmigiani and Thouless (2006) who assumed a particular choice of tensile and shear cohesive zone laws.

In order to regularize the finite element (FE) calculations, an initial stiffness $k_{\mathrm{T}}$ is specified for the cohesive elements comprising the tensile band, such that the tensile cohesive length $E/k_{\mathrm{T}}$ exceeds $20 \ell _{\mathrm{e}}$, where *E* is the reference Young’s modulus of the solid in the $x_{2}-$direction and $\ell _{e}$ is the characteristic dimension of a finite element at the notch tip in the $x_{1}-$direction. Likewise, an initial shear and normal stiffness $k_{\mathrm{S}}$ is specified for the cohesive elements comprising the shear band, such that the shear cohesive length $E/k_{\mathrm{S}}$ exceeds $20 h_{\mathrm{e}}$, where $h_{e}$ is the characteristic dimension of a finite element at the notch tip in the $x_{2}$-direction. Note that the shear band has infinite strength and infinite toughness in its opening mode; it thus acts as a bridged mode II crack. We emphasize that $E/k_{\mathrm{T}} \ll a$ and $E/k_{\mathrm{S}} \ll a$ and thus the length scale introduced by the stiffness of the cohesive elements has a negligible influence on the results presented in this paper.

## The orthotropic 2D solid

Recall that the elastic constitutive relation of a general anisotropic solid has the following vector-matrix Voigt form in the Cartesian frame $(x_{1},x_{2},x_{3})$ of Fig. 2:

4 $$\begin{aligned} \varepsilon _{i} = \sum \limits _{j=1}^{6} S_{ij} \sigma _{j}, \quad i= \text {1 to 6} \end{aligned}$$

where $\{ \varepsilon _{i} \} = \{ \varepsilon _{11}, \varepsilon _{22}, \varepsilon _{33}, {\gamma }_{23}, {\gamma }_{13}, {\gamma }_{12} \}^{T}, \{ \sigma _{i} \} = \{ \sigma _{11}, \sigma _{22}, \sigma _{33}, \tau _{23}, \tau _{13}, \tau _{12} \}^{T}$, and $\left[ S_{ij} \right]$ is a $(6 \mathrm X 6)$ compliance matrix with 12 independent constants. When the material has elastic symmetry plane normal to $x_{3}-$axis, the stress versus strain relation for deformation in the $(x_{1},x_{2})$ plane can be reduced to (Lekhnitskii et al. 1968)

5 $$\begin{aligned} \varepsilon _{i} = \sum \limits _{j=1,2,6} A_{ij} \sigma _{j} , \quad i=1, 2, 6 \end{aligned}$$

where

6 $$\begin{aligned} A_{ij} = {\left\{ \begin{array}{ll} S_{ij}, \quad \text {for plane stress} \\ S_{ij}-\dfrac{S_{i3}S_{j3}}{S_{33}}, \quad \text {for plane strain,} \quad i,j=1,2,6 \end{array}\right. } \end{aligned}$$

Further, if the material is orthotropic, with the $x_{1}$ and $x_{2}$ axes coincident with the principal axes of the material, there are only four independent constants $A_{11}$, $A_{12}=A_{21}$, $A_{22}$ and $A_{66}$, with $A_{16}=A_{26}=0$. In this case, the non-zero components $S_{ij}$ are related to the usual engineering constants by

7 $$\begin{aligned} S_{11}= & {} \dfrac{1}{E_{1}},\quad S_{22}=\dfrac{1}{E_{2}},\quad S_{33}=\dfrac{1}{E_{3}}\nonumber \\ S_{12}= & {} -\dfrac{\nu _{12}}{E_{1}}=S_{21}=-\dfrac{\nu _{21}}{E_{2}}\nonumber \\ S_{13}= & {} -\dfrac{\nu _{13}}{E_{1}}=S_{31}=-\dfrac{\nu _{31}}{E_{3}}\nonumber \\ S_{23}= & {} -\dfrac{\nu _{23}}{E_{2}}=S_{32}=-\dfrac{\nu _{32}}{E_{3}}\nonumber \\ S_{66}= & {} \dfrac{1}{G_{12}} \end{aligned}$$

**Fig. 3:**
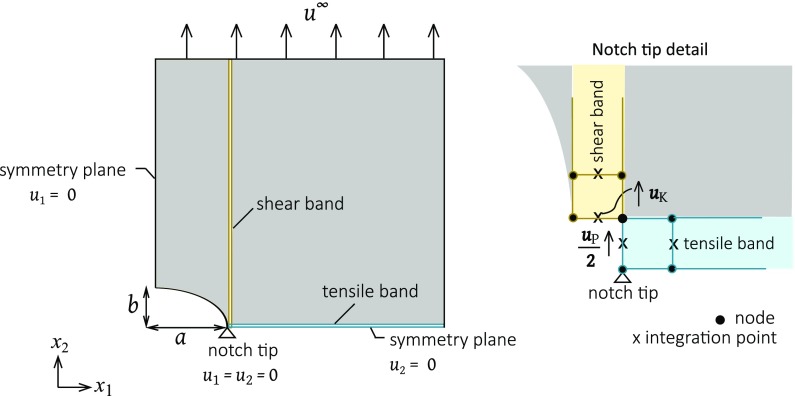
The boundary value problem and notation for crack opening displacements

Additional simplification can be achieved as follows. Suo et al. (1991) has shown that the stress state in an orthotropic solid under plane strain deformation depends only on two non-dimensional elastic parameters $\lambda$ and $\rho$ as given by

8 $$\begin{aligned} \lambda = \dfrac{A_{11}}{A_{22}} \quad \text {and} \quad \rho = \dfrac{2A_{12}+A_{66}}{2 \sqrt{A_{11}A_{22}}} \end{aligned}$$

The parameters $\lambda$ and $\rho$ quantify the degree of anisotropy. For example, $\lambda =\rho =1$ denotes isotropy and $\lambda =1$, $\rho \ne 1$ denotes transverse isotropy. Positive definiteness of the strain energy density function implies $\lambda>$0 and $\rho >-$1.

## Numerical analysis

The FE model of the problem, as defined in Fig. 2, is now described. The notch geometry and cohesive zones are sketched in Fig. 3, along with the boundary conditions to simulate uniaxial tension along the $x_{2}$ direction. FE calculations are performed using the commercial FE code ABAQUS (version 6.14). The mesh comprises 8-noded linear elements that are constrained in the out-of-plane direction to simulate plane strain ($\varepsilon _{33}=0$). The tensile band directly ahead of the crack, and the shear bands orthogonal to the initial crack are modelled by zero-thickness cohesive elements (type COH3D8); the constitutive laws as described in Sect. 1.1 are implemented by means of a user subroutine UMAT. An increasing displacement $u_{2}=u^{\infty }$ is applied to the top edge of the specimen. The resulting net section stress is $\sigma ^{\infty }= P/(W-a)$ where *P* is the sum of the reaction forces per unit thickness on the top edge. The relevant notch tip displacements are indicated in the inset of Fig. 3.

**Fig. 4:**
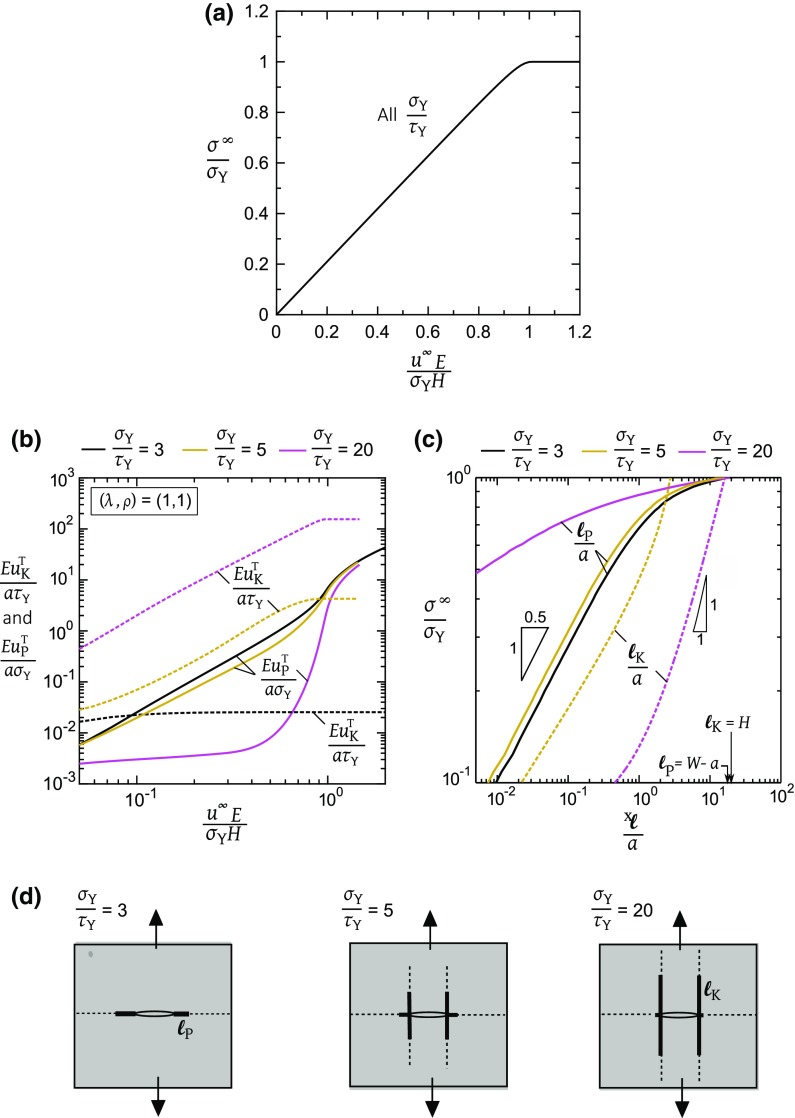
Uniaxial tensile response of a centre-crack of length 2*a* in an isotropic solid, with tensile plastic zone ahead of crack tip and shear plastic zones orthogonal to the cracking plane. The cohesive zones have infinite toughness in these simulations such that continued plastic flow occurs. **a** Net-section tensile stress $\sigma ^{\infty }$ versus remote displacement $u^{\infty }$; **b** Crack tip displacements $u_{\mathrm{P}}$ and $u_{\mathrm{K}}$ versus $u^{\infty }$; **c** $\sigma ^{\infty }$ versus plastic zone lengths, $\ell _{\mathrm{P}}$ and $\ell _{\mathrm{K}}$; **d** Plastic zone lengths $\ell _{\mathrm{P}}$ and $\ell _{\mathrm{K}}$ (for $\sigma _{\mathrm{Y}}/\tau _{\mathrm{Y}}=3, 5,$ and 20) at $\sigma ^{\infty }=0.5\sigma _{\mathrm{Y}}$ (solid lines), and at $\sigma ^{\infty }=\sigma _{\mathrm{Y}}$ (dashed lines)

It is instructive to consider the response of a centre-cracked panel with tensile and shear cohesive zones for the case $u^\mathrm{f}_{\mathrm{P}}=u^\mathrm{f}_{\mathrm{K}}=\infty$, that is for the case where continued plastic flow can occur without failure of either cohesive zone. We limit attention to the isotropic case $(\lambda , \rho )=(1,1)$ and strength ratios $\sigma _{\mathrm{Y}}/\tau _{\mathrm{Y}}=3, 5,$ and 20. The macroscopic stress $\sigma ^{\infty }$ versus displacement $u^{\infty }$ of the centre-cracked panel is plotted in Fig. 4a for the three choices of $\sigma _{\mathrm{Y}}/\tau _{\mathrm{Y}}$. The three responses are almost indistinguishable in this plot due to the small values of *a* / *H* and *a* / *W*. Predictions are shown in Fig. 4b for the tip opening displacement $u^\mathrm{T}_{\mathrm{P}}$ of the tensile cohesive zone, and for the tip sliding displacement $u^\mathrm{T}_\mathrm{K}$ for the shear cohesive zone as a function of the remote displacement $u^{\infty }$. We define the lengths $\ell _{\mathrm{P}}$ and $\ell _{\mathrm{K}}$, respectively, of the tensile and shear plastic zones as the lengths of the cohesive zone over which the displacements satisfy $u_{\mathrm{P}} \ge \sigma _{\mathrm{Y}}/k_{\mathrm{T}}$ and $u_{\mathrm{K}} \ge \tau _{\mathrm{Y}}/k_{\mathrm{S}}$. The lengths $\ell _{\mathrm{P}}$ and $\ell _{\mathrm{K}}$ are plotted in Fig. 4c, as a function of the normalised applied stress $\sigma ^{\infty }/\sigma _{\mathrm{Y}}$ for selected values of $\sigma _\mathrm{Y}/\tau _{\mathrm{Y}}$. The relative growth of the two plastic zones is sensitive to the choice of $\sigma _{\mathrm{Y}}/\tau _{\mathrm{Y}}$, and we proceed to describe this competition.

Three distinct behaviours emerge, depending on the assumed value of $\sigma _{\mathrm{Y}}/\tau _{\mathrm{Y}}$. For the choice $\sigma _\mathrm{Y}/\tau _{\mathrm{Y}}=3$, the shear cohesive zone is not activated, and only a tensile plastic zone spreads across the ligament of the panel with increasing $\sigma ^{\infty }/\sigma _{\mathrm{Y}}$. This is depicted in the sketch of $\ell _{\mathrm{P}}$ and $\ell _{\mathrm{K}}$ in Fig. 4d for $\sigma ^{\infty }/\sigma _\mathrm{Y}=0.5$ and $\sigma ^{\infty }/\sigma _{\mathrm{Y}}=1$. In contrast, for $\sigma _{\mathrm{Y}}/\tau _{\mathrm{Y}}=5$, the shear plastic zone is longer than the tensile cohesive zone under increasing $\sigma ^{\infty }$, provided $\sigma ^{\infty }/\sigma _{\mathrm{Y}} < 0.86$. A cross-over in response occurs such that $\ell _{\mathrm{P}}=\ell _{\mathrm{K}}=2.6a$ at $\sigma ^{\infty }/\sigma _{\mathrm{Y}} = 0.86$. At higher values of $\sigma ^{\infty }/\sigma _{\mathrm{Y}}$ in the range of 0.86 to unity, the tensile plastic zone spreads across the ligament while the shear plastic zone arrests, see Fig. 4c. The relative lengths of $\ell _{\mathrm{P}}$ and $\ell _{\mathrm{K}}$ at $\sigma ^{\infty }/\sigma _{\mathrm{Y}}=0.5$ and $\sigma ^{\infty }/\sigma _{\mathrm{Y}}=1$ are sketched in Fig. 4d, and this switch in behaviour is again evident.

**Fig. 5:**
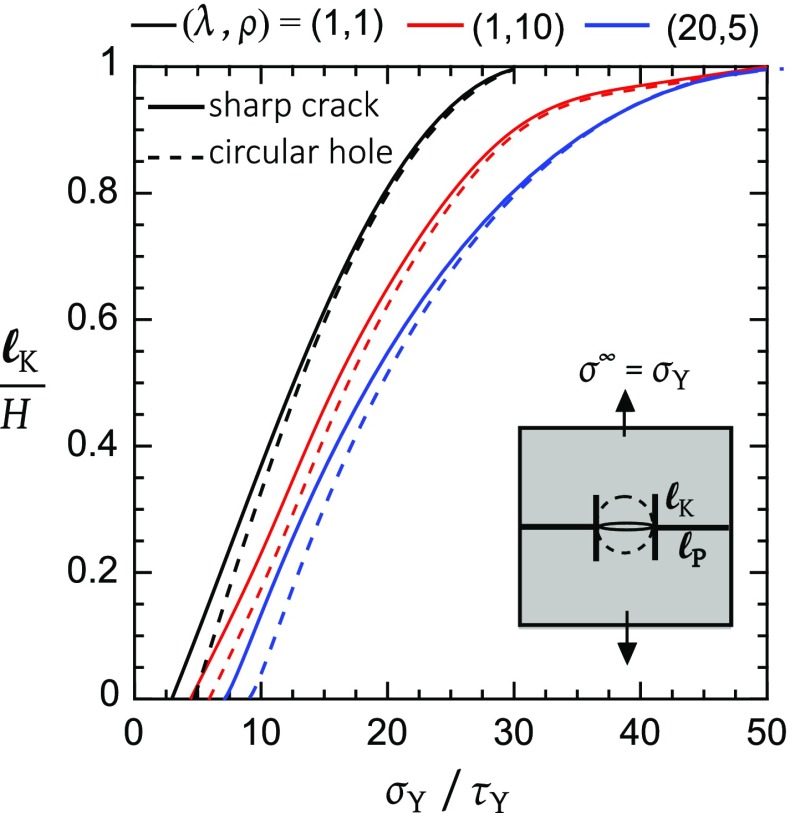
Plastic kink length $\ell _{\mathrm{K}}$ at tensile plastic collapse, $\sigma ^{\infty }=\sigma _{\mathrm{Y}}$, as a function of the strength ratio $\sigma _{\mathrm{Y}}/\tau _{\mathrm{Y}}$ for selected values of $(\lambda , \rho )$

Now consider the choice $\sigma _{\mathrm{Y}}/\tau _{\mathrm{Y}}=20$. Again, the shear plastic zone grows faster than the tensile plastic zone for $\sigma ^{\infty }/\sigma _{\mathrm{Y}}$ below a transition value; the transition is now at $\ell _{\mathrm{P}}=\ell _{\mathrm{K}}=16.2a$ for $\sigma ^{\infty }/\sigma _{\mathrm{Y}} = 0.998$. Subsequently, the tensile plastic zone sweeps across the ligament of the panel with arrest of the shear plastic zone. This is sketched in Fig. 4d. It is clear from Fig. 4d that the extent of the shear plastic zone at the point of collapse (i.e., $\sigma ^\infty /\sigma _\mathrm{Y} =1)$ is sensitive to the choice of $\sigma _{\mathrm{Y}}/\tau _{\mathrm{Y}}$. This is shown explicitly in Fig. 5 by plotting $\ell _{\mathrm{K}}/H$ at $\sigma ^{\infty }/\sigma _{\mathrm{Y}}=1$ versus $\sigma _{\mathrm{Y}}/\tau _{\mathrm{Y}}$. Shear yielding is not triggered in the isotropic solid provided $\sigma _{\mathrm{Y}}/\tau _{\mathrm{Y}} \le 3$, as discussed in Tankasala et al. (2017). As $\sigma _{\mathrm{Y}}/\tau _\mathrm{Y}$ is increased from 3 to 30, $\ell _{\mathrm{K}}/H$ increases from zero to 1. At higher $\sigma _{\mathrm{Y}}/\tau _{\mathrm{Y}}$, $\ell _{\mathrm{K}}/H$ remains at this saturated value.

For completeness, the sensitivity of the magnitude of $\ell _\mathrm{K}/H$ at $\sigma ^{\infty }/\sigma _{\mathrm{Y}}=1$ to the choice of $\sigma _{\mathrm{Y}}/\tau _{\mathrm{Y}}$ is shown in Fig. 5 for the centre crack and for the circular hole in an isotropic solid. Only a small sensitivity to response is evident. For the circular hole, no shear plastic zone is activated for $\sigma _\mathrm{Y}/\tau _{\mathrm{Y}} \le 4.5$; at larger $\sigma _{\mathrm{Y}}/\tau _{\mathrm{Y}}$ exceeding this threshold value, $\ell _{\mathrm{K}}/H$ increases with increasing $\sigma _{\mathrm{Y}}/\tau _{\mathrm{Y}}$ until it attains a value of near unity at $\sigma _{\mathrm{Y}}/\tau _{\mathrm{Y}}=30$. The effect of material orthotropy upon the development of the shear plastic zone at $\sigma ^{\infty }/\sigma _{\mathrm{Y}}=1$ is also assessed in Fig. 5 for the case of a crack and a circular hole. We select the following 3 prototypical orthotropic solids to motivate representative values for $(\lambda , \rho )$:

1. An isotropic solid with $(\lambda ,\rho )=(1,1)$. This is relevant to the case of a quasi-isotropic composite in which the elastic constants of fibres and matrix are almost identical, such as SiC–SiC ceramic-matrix composites.
2. An intermediate orthotropic solid with $(\lambda ,\rho )=(1,10)$ represents a composite with equal direct moduli in the $0^{\circ }$ and $90^{\circ }$ directions, but a reduced shear modulus. Oxide-oxide ceramic matrix composites are of this type.
3. A strongly orthotropic solid with $(\lambda ,\rho )=(20,5)$ of reduced shear modulus: a unidirectional CFRP laminate is an example of such a solid.

First, note from Fig. 5 that an increase in $\rho$ or $\lambda$ gives rise to a small drop in value of $\ell _{\mathrm{K}}/H$ at fixed $\sigma _{\mathrm{Y}}/\tau _{\mathrm{Y}}$. This can be interpreted as follows: an increase in the relative transverse compliance $E_{1}/E_{2}$ or shear compliance $G_{12}/E_{2}$ reduces the requirement of a long shear plastic zone on compatibility grounds. Second, the value of $\ell _{\mathrm{K}}/H$ at $\sigma ^{\infty }/\sigma _{\mathrm{Y}}=1$ is only sensitive to the choice of hole versus crack when $\ell _{\mathrm{K}}/H$ is small, which typically occurs for $\sigma _{\mathrm{Y}}/\tau _{\mathrm{Y}}$ below 20.

## Predictions for the notch tensile strength

Recall from the Introduction that the mode of fracture and consequently the corresponding net-section tensile strength of the notched panel are governed by the attainment of a critical value of $u_{\mathrm{P}}^\mathrm{T}=u_{\mathrm{P}}^\mathrm{f}$ or $u_{\mathrm{K}}^\mathrm{T}=u_\mathrm{K}^\mathrm{f}$. Accordingly, we define the tensile strength of the panel $\sigma ^{\infty }_{\mathrm{f}}$ as the net-section stress $\sigma ^{\infty }$ corresponding to the first attainment of $u_\mathrm{P}^\mathrm{f}$ or $u_{\mathrm{K}}^\mathrm{f}$. The predictions of $\sigma ^{\infty }_{\mathrm{f}}$ for a panel containing a sharp crack or a circular hole are discussed in turn. In each case, the role of orthotropy is investigated using the above selected values of $\lambda$ and $\rho$ to represent three classes of composites.

### Panel containing a sharp crack

First consider the case of a sharp crack of semi-length *a*. We know from Tankasala et al. (2017) that a tensile plastic zone exists ahead of the tip of a long crack for all values of $\sigma _\mathrm{Y}/\tau _{\mathrm{Y}} \ge 0$. However, plasticity within the shear band is activated only for $\sigma _{\mathrm{Y}}/\tau _{\mathrm{Y}}>\sigma _\mathrm{Y}/\tau _{\mathrm{L}}$, where the transition value $\sigma _\mathrm{Y}/\tau _{\mathrm{L}}$ depends on the values of $(\lambda ,\rho )$ for the solid. For the three cases of orthotropy considered here, our current calculations predict transition values for a finite crack ($a/W=0.05$, $W/H=1$) as $\sigma _{\mathrm{Y}}/\tau _{\mathrm{L}}=3$ for $(\lambda , \rho )=(1,1)$, $\sigma _{\mathrm{Y}}/\tau _{\mathrm{L}}=4.5$ for $(\lambda , \rho )=(1,10)$, and $\sigma _{\mathrm{Y}}/\tau _{\mathrm{L}}=7.8$ for $(\lambda , \rho )=(20,5)$. The tensile strength of the cracked panel in the presence of active tensile and shear plastic zones is dictated by the zone that fails first. Accordingly, two modes of failure are possible:

(i) The **penetration** mode is active when the tensile band fails first: $u_{\mathrm{P}}$ attains the value $u_{\mathrm{P}}^\mathrm{f}$, resulting in crack growth along $x_{1}$ direction of Fig. 2. Dimensional analysis dictates that the notch tensile strength is given by
  9 $$\begin{aligned} \dfrac{\sigma ^{\infty }_{\mathrm{f}}}{\sigma _{\mathrm{Y}}}=f_{\mathrm{1}} \left( \dfrac{a \sigma _{\mathrm{Y}}^{2}}{E \mathrm {\Gamma }_{\mathrm{P}}}, \dfrac{a}{W}, \dfrac{W}{H}, \dfrac{\sigma _{\mathrm{Y}}}{\tau _{\mathrm{Y}}}, \lambda , \rho \right) \end{aligned}$$
  where the reference modulus *E* is defined as $E=1/A_{22}$.
(ii) The **kinking** mode occurs when the shear band fails first: $u_{\mathrm{K}}$ attains the value $u_{\mathrm{K}}^\mathrm{f}$, causing splitting along the $x_{2}$ direction of Fig. 2. The notch tensile strength for this mode of failure reads
  10 $$\begin{aligned} \dfrac{\sigma ^{\infty }_{\mathrm{f}}}{\tau _{\mathrm{Y}}}=f_{\mathrm{2}} \left( \dfrac{a \tau _{\mathrm{Y}}^{2}}{E \mathrm {\Gamma }_{\mathrm{K}}}, \dfrac{a}{W}, \dfrac{W}{H}, \dfrac{\sigma _{\mathrm{Y}}}{\tau _{\mathrm{Y}}}, \lambda , \rho \right) \end{aligned}$$

**Fig. 6:**
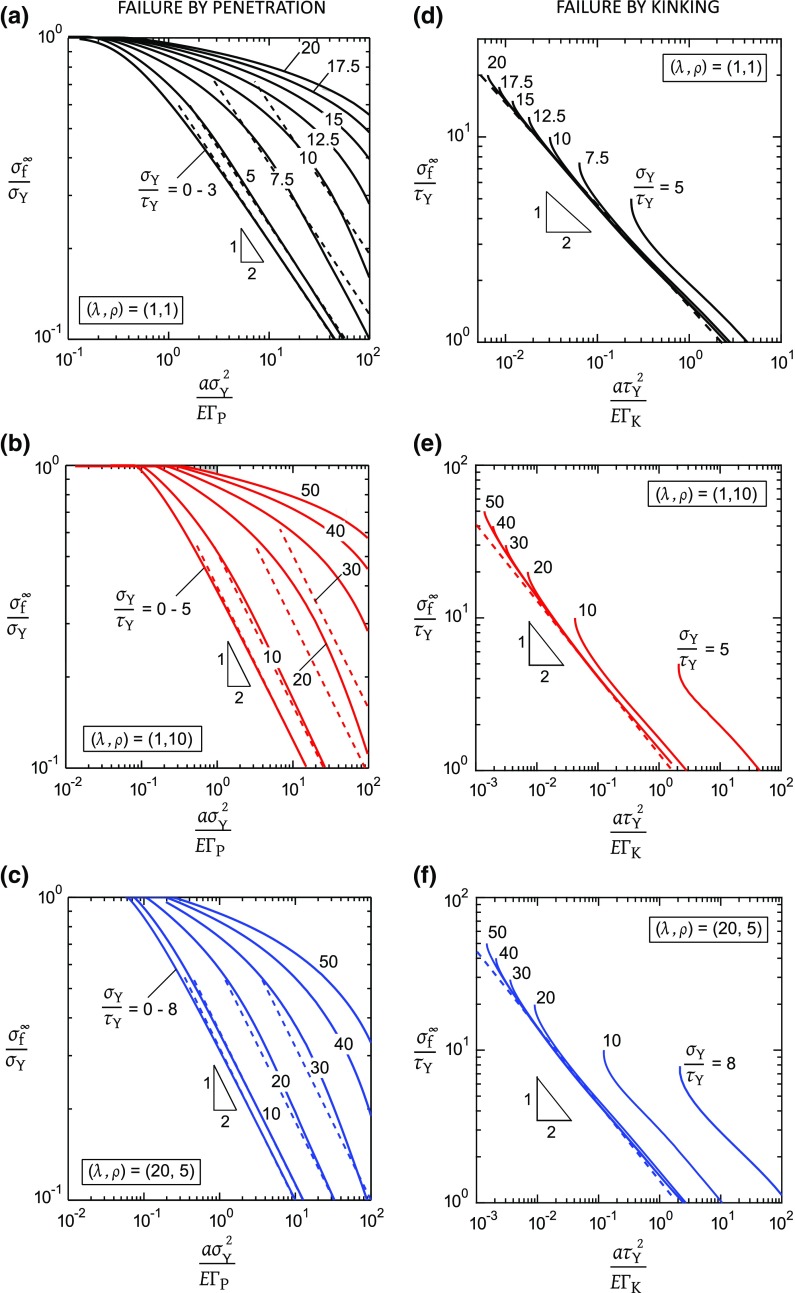
Notch tensile strength for failure by penetration (**a**–**c**) or by kinking (**d**–**f**) from the tip of a sharp pre-crack: **a**, **d** isotropic solid, $(\lambda , \rho )=(1,1)$; **b**, **e** orthotropic solid, $(\lambda , \rho )=(1,10)$; **c**, **f** orthotropic solid, $(\lambda , \rho )=(20,5)$. The dashed lines are the long crack limit

The non-dimensional functions $f_{\mathrm{1}}$ and $f_{\mathrm{2}}$ are obtained from the FE simulations for a wide range of $\sigma _\mathrm{Y}/\tau _{\mathrm{Y}}$ between 0 and 60, and these are plotted in Fig. 6 for selected values of orthotropy ($\lambda , \rho$) (and for $a/W=a/H=0.05$). We first assume that failure is by penetration or by kinking, and then in Sect. 6 develop failure mechanism maps that detail their competition.

Consider first the predictions for notch strength when penetration is the active mode of failure. The results shown in Fig. 6a–c for the 3 values of orthotropy are qualitatively similar. For $0<\sigma _\mathrm{Y}/\tau _{\mathrm{Y}}<\sigma _{\mathrm{Y}}/\tau _{\mathrm{L}}$, only a tensile plastic zone exists ahead of the crack with no triggering of the shear zones. This response is the well-known Dugdale solution. In contrast, for $\sigma _{\mathrm{Y}}/\tau _{\mathrm{Y}}>\sigma _{\mathrm{Y}}/\tau _\mathrm{L}$, additional shielding of the crack-tip is provided by the presence of shear plastic zones, each of length $\ell _{\mathrm{K}}^\mathrm{f}$ aligned with the loading direction in addition to the tensile plastic zone, and consequently the notch strength is elevated. At small values of $\tau _{\mathrm{Y}}$, such as $\sigma _{\mathrm{Y}}/\tau _\mathrm{Y}>10$ for the isotropic case, the active shear bands are much longer than the tensile plastic zone at the onset of failure by crack penetration. A physical interpretation is that the active shear bands reduce the stress concentration on the tensile band, thereby “blunting” the tensile crack. Thus, ${\sigma ^{\infty }_{\mathrm{f}}}/{\sigma _{\mathrm{Y}}}$ increases with increasing $\sigma _{\mathrm{Y}}/\tau _{\mathrm{Y}}$, as shown in Fig. 6a–c, for any given value of ${a \sigma _{\mathrm{Y}}^{2}}/{E \mathrm {\Gamma }_{\mathrm{P}}}$. We note that ${\sigma ^{\infty }_{\mathrm{f}}}/{\sigma _{\mathrm{Y}}}$ scales as $({a \sigma _{\mathrm{Y}}^{2}}/{E \mathrm {\Gamma }_{\mathrm{P}}})^{-1/2}$ for ${\sigma ^{\infty }_{\mathrm{f}}}/{\sigma _{\mathrm{Y}}}<0.4$ in the regime $\sigma _{\mathrm{Y}}/\tau _{\mathrm{Y}}<5$ and for all choices of $(\lambda , \rho )$. This corresponds to the long crack case where linear elastic fracture mechanics (LEFM) applies and a *K*-field exists in the vicinity of the crack tip. In this regime, the failure strength $\sigma ^{\infty }_{\mathrm{f}}$ can be written in terms of a *penetration fracture toughness* $K_{\mathrm{IC, P}}$ such that

11 $$\begin{aligned} \sigma ^{\infty }_{\mathrm{f}} = \dfrac{K_{\mathrm{IC, P}}}{\sqrt{\pi a}} \quad Y \left( \dfrac{a}{W} \right) \end{aligned}$$

where

12 $$\begin{aligned} K_{\mathrm{IC, P}}^{2} = c_{1} \left( \lambda , \rho \right) \, EG_\mathrm{f}^{\infty } \end{aligned}$$

is the usual Irwin-type relationship relating the mode I stress intensity factor $K_{\mathrm{P}}$ to the energy release rate $G^{\infty }$. The geometric *K*-calibration factor *Y*(*a* / *W*) on the right side of (11) has the value $Y=1.0012$ for $a/W=0.05$, see for example Tada et al. (1985). The constant $c_{1} \left( \lambda , \rho \right)$ is given by $c_{1}=\lambda ^{-1/4} \sqrt{{2}/{(1+\rho )}}$ for a crack aligned with the $x_{1}$ direction of Fig. 2 (Sih et al. 1965). Now, Tankasala et al. (2017) have obtained $G_\mathrm{f}^{\infty }/\mathrm {\Gamma }_{\mathrm{P}}$ for a semi-infinite crack as a function of $\sigma _{\mathrm{Y}}/\tau _{\mathrm{Y}}$ for the same 3 choices of orthotropy $(\lambda , \rho )$ as considered here, see Fig. 5(a) of Tankasala et al. (2017). The long crack prediction for ${\sigma ^{\infty }_{\mathrm{f}}}/{\sigma _{\mathrm{Y}}}$ follows from (11) and (12). This asymptote has been added to Fig. 6a–c of the present study for selected values of $\sigma _\mathrm{Y}/\tau _{\mathrm{Y}}$. We note that the minimum value of ${a \sigma _\mathrm{Y}^{2}}/{E \mathrm {\Gamma }_{\mathrm{P}}}$ for which the long crack prediction, i.e., small scale yielding (SSY) is accurate, increases with increasing $\sigma _{\mathrm{Y}}/\tau _{\mathrm{Y}}$. This is traced to the fact that the SSY asymptote requires $a \gg \ell _{\mathrm{P}}$ and $a \gg \ell _{\mathrm{K}}$. However, $\ell _{\mathrm{K}}/\ell _{\mathrm{P}}$ increases dramatically with increasing $\sigma _{\mathrm{Y}}/\tau _{\mathrm{Y}}$, see Fig. 5(a) of Tankasala et al. (2017). Consequently, the requirement $a/\ell _{\mathrm{K}} \gg 1$ places a severe restriction on the SSY prediction at large $\sigma _{\mathrm{Y}}/\tau _{\mathrm{Y}}$. We further note that in the long crack regime, the value of ${\sigma ^{\infty }_\mathrm{f}}/{\sigma _{\mathrm{Y}}}$ at any given value of ${a \sigma _\mathrm{Y}^{2}}/{E \mathrm {\Gamma }_{\mathrm{P}}}$ increases by almost an order of magnitude when $\sigma _{\mathrm{Y}}/\tau _{\mathrm{Y}}$ increases from 3 to 20, for the isotropic case of Fig. 6a.

Now consider the alternative mode of failure, that of kinking by progressive rupture of the shear band. The FE estimates of notch tensile strength for kinking are plotted in Fig. 6d–f. Recall that a shear plastic zone only exists for $\tau _{\mathrm{Y}}<\tau _{\mathrm{L}}$ where $\sigma _{\mathrm{Y}}/\tau _{\mathrm{L}}$ depends upon $(\lambda ,\rho )$ of the solid. For the isotropic case of Fig. 6d, we have already observed that $\sigma _{\mathrm{Y}}/\tau _{\mathrm{L}} = 3$ and consequently no shear plastic zone exists for $\sigma _\mathrm{Y}/\tau _{\mathrm{Y}}=3$. The dependence of ${\sigma ^{\infty }_\mathrm{f}}/{\tau _{\mathrm{Y}}}$ upon $({a \tau _{\mathrm{Y}}^{2}}/{E \mathrm {\Gamma }_{\mathrm{K}}})$ is shown for $\sigma _{\mathrm{Y}}/\tau _\mathrm{Y}\ge 5$. For a sufficiently large $\sigma _{\mathrm{Y}}/\tau _{\mathrm{Y}}$, such as $\sigma _{\mathrm{Y}}/\tau _{\mathrm{Y}}>7.5$, ${\sigma ^{\infty }_\mathrm{f}}/{\tau _{\mathrm{Y}}}$ is only slightly influenced by the value of $\sigma _{\mathrm{Y}}/\tau _{\mathrm{Y}}$, implying that the presence of a tensile plastic zone has only a minor effect upon the kinking strength ${\sigma ^{\infty }_{\mathrm{f}}}/{\tau _{\mathrm{Y}}}$. This is in contrast to the failure response of the tensile cohesive zone, recall Fig. 6a.

Next, consider the orthotropic case of Fig. 6e. For the choice $(\lambda , \rho )=(1,10)$ and $\sigma _{\mathrm{Y}}/\tau _{\mathrm{Y}}=5$, the value of strength ratio $\sigma _{\mathrm{Y}}/\tau _{\mathrm{Y}}$ only just exceeds the limiting value of $\sigma _{\mathrm{Y}}/\tau _{\mathrm{L}}$: a short shear plastic zone exists in relation to the tensile plastic zone. In this regime, ${\sigma ^{\infty }_{\mathrm{f}}}/{\tau _{\mathrm{Y}}}$ is sensitive to the magnitude of $\sigma _{\mathrm{Y}}/\tau _{\mathrm{Y}}$. At higher values of $\sigma _{\mathrm{Y}}/\tau _{\mathrm{Y}}$, the shear plastic zone is sufficiently long in relation to the tensile plastic zone for the tensile cohesive zone to have only a minor effect upon the kinking failure. Consequently, ${\sigma ^{\infty }_{\mathrm{f}}}/{\tau _{\mathrm{Y}}}$ is almost insensitive to the magnitude of $\sigma _{\mathrm{Y}}/\tau _\mathrm{Y}$. A similar qualitative behaviour is noted in Fig. 6f for the case of $(\lambda , \rho )=(20, 5)$, but a more detailed discussion is omitted for the sake of brevity.

In broad terms, the macroscopic strength for failure by kinking scales as $({a \tau _{\mathrm{Y}}^{2}}/{E \mathrm {\Gamma }_\mathrm{K}})^{-1/2}$ provided ${\sigma ^{\infty }_{\mathrm{f}}}/{\tau _{\mathrm{Y}}}<10$. This behaviour is observed for all three choices of orthotropy and for all $\sigma _{\mathrm{Y}}/\tau _{\mathrm{Y}}$ (unless $\sigma _\mathrm{Y}/\tau _{\mathrm{Y}}$ is close in value to $\sigma _{\mathrm{Y}}/\tau _\mathrm{L}$). This response corresponds to the long crack solution, and a crack *kinking fracture toughness* $K_{\mathrm{IC, K}}$ can again be identified such that

13 $$\begin{aligned} \sigma ^{\infty }_{\mathrm{f}} = \dfrac{K_{\mathrm{IC, K}}}{\sqrt{\pi a}} \quad Y \left( \dfrac{a}{W} \right) \end{aligned}$$

Recall that the value of $K_{\mathrm{IC, K}}$ is related to the macroscopic mode I toughness $G_{\mathrm{f}}^{\infty }$ by the Irwin relationship

14 $$\begin{aligned} K_{\mathrm{IC, K}}^{2} = c_{2} \left( \lambda , \rho \right) \, EG_\mathrm{f}^{\infty } \end{aligned}$$

where $c_{2} \left( \lambda , \rho \right)$ is given by $c_{2}=c_{1} \sqrt{\lambda }$ for a crack aligned with the $x_{2}$ direction of Fig. 2, see Sih et al. (1965). For a semi-infinite crack, Tankasala et al. (2017) have plotted $G_\mathrm{f}^{\infty }/\mathrm {\Gamma }_{\mathrm{K}}$ versus $\sigma _\mathrm{Y}/\tau _{\mathrm{Y}}$ in Fig. 5(b) of their paper: the ratio $G_\mathrm{f}^{\infty }/\mathrm {\Gamma }_{\mathrm{K}}$ is only moderately sensitive to the magnitude of $(\lambda , \rho )$ and is almost insensitive to the value of $\sigma _{\mathrm{Y}}/\tau _{\mathrm{Y}}$ provided it is larger than about 10. The relation (13) is plotted in dashed lines in Fig. 6d–f upon taking the asymptotic value for $K_{\mathrm{IC, K}}$ for each $(\lambda , \rho )$ from Tankasala et al. (2017). We find that our results for the finite crack are consistent with the long crack case provided ${a \tau _{\mathrm{Y}}^{2}}/{E \mathrm {\Gamma }_{\mathrm{K}}}$ is sufficiently large for LEFM conditions to prevail.

### Panel containing a hole

Now consider the case of a circular hole of radius *a* in a panel made from an orthotropic solid, as shown in Fig. 2. The hole is small compared to *W* and *H* by taking $a/W=0.05$ and $W/H=1$. In the absence of cohesive zones, the maximum tensile stress at the tip of the hole scales with the remote stress $\sigma ^{\infty }$ as $\sigma _{22}(a,0)=K_\mathrm{T}\sigma ^{\infty }$, where $K_{\mathrm{T}}$ is the elastic stress concentration factor. This stress concentration factor $K_{\mathrm{T}}$ depends upon the elastic orthotropy constants $\lambda$ and $\rho$, as (Lekhnitskii 1981; Suo et al. 1991)

15 $$\begin{aligned} K_{\mathrm{T}} = 1+ \sqrt{2} \lambda ^{1/4} (1+\rho )^{1/2} \end{aligned}$$

For example, the relation (15) gives $K_{\mathrm{T}}=3$ for $(\lambda , \rho )=(1,1)$, $K_{\mathrm{T}}=5.7$ for $(\lambda , \rho )=(1,10)$, and $K_{\mathrm{T}}=7.5$ for $(\lambda , \rho )=(20,5)$. Plasticity within the tensile band at the edge of the hole is activated when the applied stress $\sigma ^{\infty }$ exceeds $\sigma _{\mathrm{Y}}/K_{\mathrm{T}}$. Unlike the case of a sharp crack where all components of stress $\sigma _{ij}$ are singular at the crack tip, the presence of a traction-free surface of a hole leads to $\sigma _{12}=0$ at the edge of the hole. Thus, the shear plastic zone does not initiate on the mid-plane *at* the edge of the hole $(x_{1}, x_{2})=(a,0)$, but rather at a small height $(\approx 0.012a)$ above (and below) the mid-plane. We find from the FE simulations that the limiting value of strength ratio $\sigma _{\mathrm{Y}}/\tau _{\mathrm{L}}$ below which plasticity does not activate within the shear band is higher for a circular hole than for a sharp crack: $\sigma _\mathrm{Y}/\tau _{\mathrm{L}}$ increases from 3 to 4.5 for $(\lambda , \rho )=(1,1)$, $\sigma _{\mathrm{Y}}/\tau _{\mathrm{L}}$ increases from 4.5 to 9.2 for $(\lambda , \rho )=(1,10)$, and $\sigma _{\mathrm{Y}}/\tau _{\mathrm{L}}$ increases from 7.8 to 11.5 for $(\lambda , \rho )=(20,5)$.

**Fig. 7:**
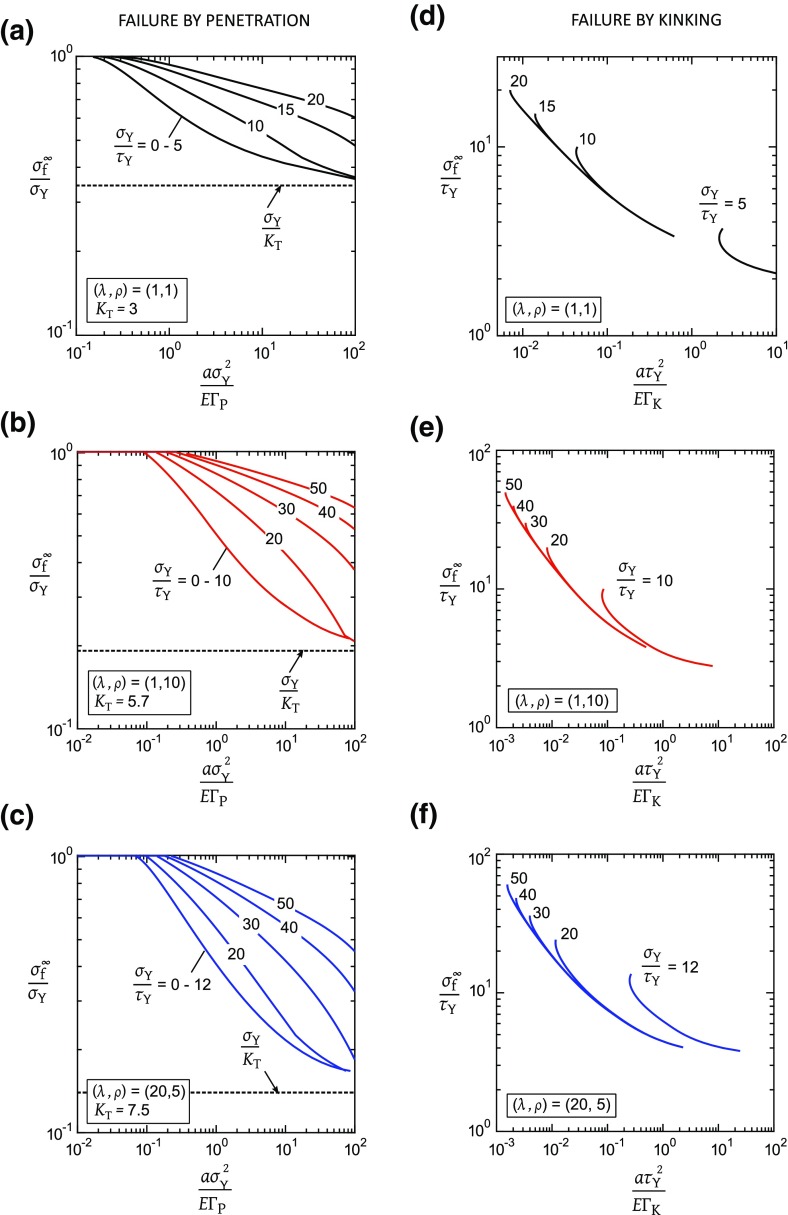
Notch tensile strength for failure by penetration (**a**–**c**) or by kinking (**d**–**f**) from the edge of a circular hole: **a**, **d** isotropic solid, $(\lambda , \rho )=(1,1)$; **b**, **e** orthotropic solid, $(\lambda , \rho )=(1,10)$; **c**, **f** orthotropic solid, $(\lambda , \rho )=(20,5)$

Two modes of failure are again considered for the tensile strength of a panel containing a circular hole: penetration along the tensile plastic zone leading to crack growth along $x_{1}$ direction, and kinking along the shear plastic zone leading to orthogonal splits in the $x_{2}$ direction. The dependence of ${\sigma ^{\infty }_\mathrm{f}}/{\sigma _{\mathrm{Y}}}$ and of ${\sigma ^{\infty }_{\mathrm{f}}}/{\tau _\mathrm{Y}}$ upon geometry and material properties for the penetration and kinking modes of failure are similar to (9) and (10), respectively, and are obtained from FE simulations performed over a range of $\sigma _{\mathrm{Y}}/\tau _{\mathrm{Y}}$ between 0 and 50. Figure 7 shows the FE predictions of notch tensile strength for the three cases of orthotropy; Fig. 7a–c assume failure by penetration whereas Fig. 7d–f assume failure by kinking.

**Fig. 8:**
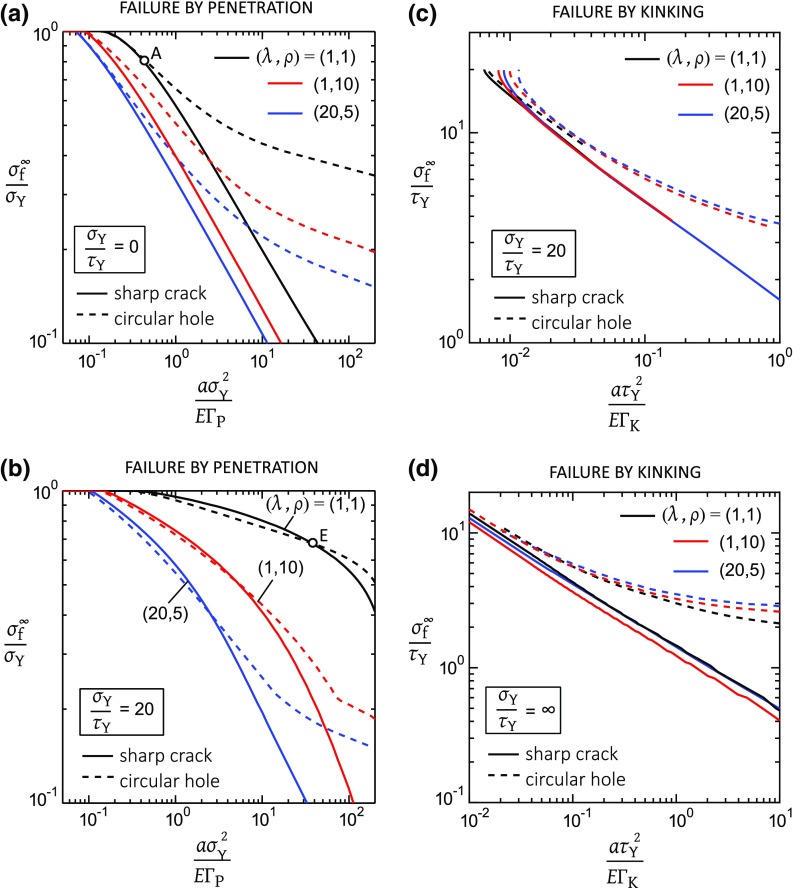
Comparison of the notch tensile strength for a sharp pre-crack and circular hole, for three choices of orthotropy. **a** $\sigma _{\mathrm{Y}}/\tau _{\mathrm{Y}}=0$ for failure by penetration, **b** $\sigma _{\mathrm{Y}}/\tau _{\mathrm{Y}}=20$ for failure by penetration, **c** $\sigma _{\mathrm{Y}}/\tau _\mathrm{Y}=20$ for failure by kinking, and **d** $\sigma _\mathrm{Y}/\tau _{\mathrm{Y}}=\infty$ for failure by kinking

Consider first the case where penetration is the critical mode of failure (such that the tensile band fails first), see Fig. 7a for example. For a sufficiently low value of $\sigma _{\mathrm{Y}}/\tau _{\mathrm{Y}}$, below a limiting value $\sigma _{\mathrm{Y}}/\tau _{\mathrm{L}}$, no shear plastic zone exists. Thus, the curves for $0 \le \sigma _{\mathrm{Y}}/\tau _{\mathrm{Y}} \le 5$ superpose for the isotropic case. A similar behaviour is noted for the two orthotropic cases of Fig. 7b, c. The notch strength $\sigma ^{\infty }_{\mathrm{f}}$ for $0 \le \sigma _\mathrm{Y}/\tau _{\mathrm{Y}} \le \sigma _{\mathrm{Y}}/\tau _{\mathrm{L}}$ increases from the notch-brittle limit $\sigma _{\mathrm{Y}}/K_{\mathrm{T}}$ to the plastic collapse limit $\sigma _{\mathrm{Y}}$ with diminishing normalized hole radius $a\sigma _{\mathrm{Y}}^{2}/E \mathrm {\Gamma }_{\mathrm{P}}$: the tensile plastic zone “blunts” the elastic stress concentration. As $a\sigma _{\mathrm{Y}}^{2}/E \mathrm {\Gamma }_{\mathrm{P}}$ decreases, we find that $\ell _{\mathrm{P}}^\mathrm{f}/a$ increases and $\sigma ^{\infty }_\mathrm{f}$ approaches $\sigma _{\mathrm{Y}}$, in the expected manner of a “Dugdale crack”. This finding is qualitatively consistent with the widely-documented *hole size effect* observed in experiments on fibre-reinforced composites: the extent of “damage” ahead of the hole relative to the hole radius *a* increases as *a* decreases (Iarve et al. 2005; Green et al. 2007; Wisnom 2012). Additional strengthening accompanies the presence of shear plastic zones at $\sigma _{\mathrm{Y}}/\tau _{\mathrm{Y}}>\sigma _{\mathrm{Y}}/\tau _{\mathrm{L}}$: the shear plastic zones shield the tensile plastic zone and thereby elevate $\sigma ^{\infty }_{\mathrm{f}}/\sigma _{\mathrm{Y}}$.

Now consider the alternative failure mechanism of kinking, see Fig. 7d–f. The behaviour is qualitatively similar to that of a sharp crack, such that ${\sigma ^{\infty }_{\mathrm{f}}}/{\tau _{\mathrm{Y}}}$ scales with $({a \tau _{\mathrm{Y}}^{2}}/{E \mathrm {\Gamma }_{\mathrm{K}}})$ and is almost independent of $\sigma _{\mathrm{Y}}/\tau _{\mathrm{Y}}$ provided it is sufficiently high. In this regime, the tensile plastic zone is small compared to the shear plastic zone at the onset of kinking failure and has only a small influence on ${\sigma ^{\infty }_\mathrm{f}}/{\tau _{\mathrm{Y}}}$.

### Comparison of the notch geometries

It is instructive to compare the notch tensile strength for a sharp crack and for a circular hole for various degrees of orthotropy, see Fig. 8. We limit attention to three cases, $\sigma _{\mathrm{Y}}/\tau _{\mathrm{Y}}=0, 20$, and $\infty$. For the choice $\sigma _{\mathrm{Y}}/\tau _{\mathrm{Y}}=0$, only a tensile cohesive zone is present and kinking cannot occur, see Fig. 8a. In general, the strength ${\sigma ^{\infty }_{\mathrm{f}}}/\sigma _{\mathrm{Y}}$ for a circular hole of radius *a* exceeds that for a sharp crack of semi-length *a*. However, at small $a \sigma _{\mathrm{Y}}^{2}/E \mathrm {\Gamma }_{\mathrm{P}}$, the precise notch geometry becomes irrelevant as the tensile cohesive zone at the onset of penetration is much longer than the notch size, for all three values of $(\lambda , \rho )$ considered.

**Fig. 9:**
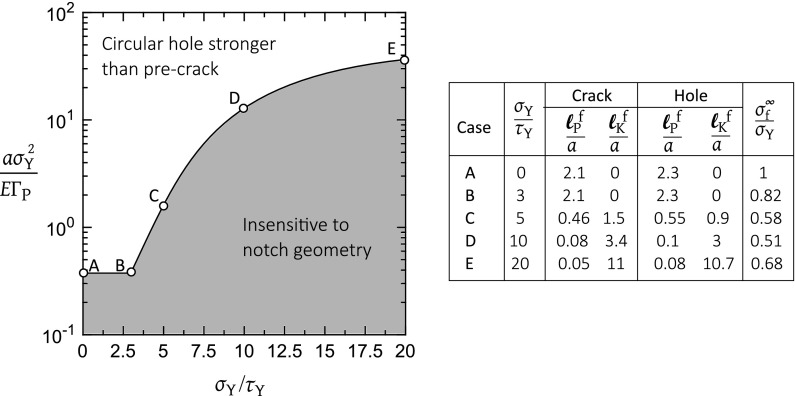
Notch sensitivity for penetration failure of an isotropic solid

Second, consider $\sigma _{\mathrm{Y}}/\tau _{\mathrm{Y}}=20$. Then, failure can occur either by penetration or by kinking, see Fig. 8b, c, respectively. For the case of penetration, the strength ${\sigma ^{\infty }_\mathrm{f}}/{\sigma _{\mathrm{Y}}}$ for the sharp crack is less than that for the circular hole at large $a \sigma _{\mathrm{Y}}^{2}/E \mathrm {\Gamma }_\mathrm{P}$, consistent with the results in Fig. 8a for which a shear plastic zone is absent. The strength ${\sigma ^{\infty }_{\mathrm{f}}}/\sigma _{\mathrm{Y}}$ for the hole and crack are comparable for sufficiently small $a \sigma _{\mathrm{Y}}^{2}/E \mathrm {\Gamma }_{\mathrm{P}}$ below a transition value, with the transition size sensitive to the degree of orthotropy. The regime in $(\sigma _{\mathrm{Y}}/\tau _{\mathrm{Y}},a \sigma _{\mathrm{Y}}^{2}/E \mathrm {\Gamma }_{\mathrm{P}})$ space for which the two geometries have a comparable notch strength due to failure by penetration is plotted in Fig. 9 for the isotropic solid. The boundary is defined by the arbitrary choice that ${\sigma ^{\infty }_{\mathrm{f}}}/{\sigma _{\mathrm{Y}}}$ for the hole and pre-crack are in agreement to within 2%. Selected choices of $\sigma _{\mathrm{Y}}/\tau _{\mathrm{Y}}$ are labelled A to E in Fig. 9 and are included within the figure in a tabulated form. Point A is the case $\sigma _{\mathrm{Y}}/\tau _{\mathrm{Y}}=0$ and $a \sigma _{\mathrm{Y}}^{2}/E \mathrm {\Gamma }_{\mathrm{P}}=0.38$, and this choice is included in Fig. 8a. Now, the shear plastic zone vanishes for $0 \le \sigma _{\mathrm{Y}}/\tau _\mathrm{Y} \le 3$ for the isotropic solid, and the limiting case of $\sigma _{\mathrm{Y}}/\tau _{\mathrm{Y}}=3$ is labelled point B in Fig. 9. For both points A and B, the tensile plastic zone at failure has a length $\ell _{\mathrm{P}}^\mathrm{f}=2.1a$ for the crack and a comparable length $\ell _{\mathrm{P}}^\mathrm{f}=2.3a$ for the hole, as tabulated in Fig. 9. As $\sigma _\mathrm{Y}/\tau _{\mathrm{Y}}$ increases from 3 to 20, the transition value of $a \sigma _{\mathrm{Y}}^{2}/E \mathrm {\Gamma }_{\mathrm{P}}$ increases as shown in Fig. 9. The length of shear plastic zone $\ell _{\mathrm{K}}^\mathrm{f}$ increases and the length $\ell _{\mathrm{P}}^\mathrm{f}$ of the tensile plastic zone drops with increasing $\sigma _\mathrm{Y}/\tau _{\mathrm{Y}}$, as shown by points C to E in Fig. 9 and the associated table. (For completeness, point E is included in Fig. 8b.)

Now consider failure by kinking, with $\sigma _{\mathrm{Y}}/\tau _\mathrm{Y}=20$ in Fig. 8c and $\sigma _\mathrm{Y}/\tau _{\mathrm{Y}}=\infty$ in Fig. 8d. For all three choices of $(\lambda , \rho )$ and the full range of $a \tau _{\mathrm{Y}}^{2}/E \mathrm {\Gamma }_{\mathrm{K}}$, the notch strength ${\sigma ^{\infty }_{\mathrm{f}}}$ for a circular hole exceeds that for a sharp crack. Also, the degree of orthotropy has only a minor effect upon the notch strength due to kinking for both the circular hole and the sharp crack.

## Failure mechanism maps

**Fig. 10:**
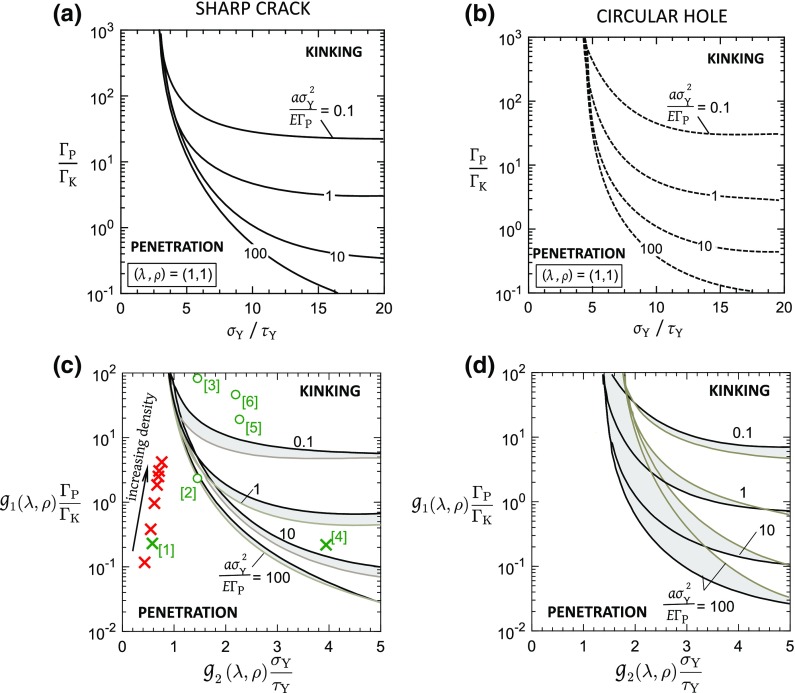
Failure mechanism maps showing the regimes of dominance of tensile failure by penetration and shear failure by kinking. Isotropic solid with a **a** centre crack of semi-length *a*, **b** circular hole of radius *a*. The corresponding maps for anisotropic solids with **c** centre crack and **d** circular hole. In (**c**, **d**), the black lines correspond to the calculations for $(\lambda , \rho )=(1,1)$ while the gray lines are for an anisotropic solid with $(\lambda , \rho )=(20,5)$. In (**c**), experimental data for woods (red) and ceramic composites (green) are included with X and O indicating the observed failure modes of penetration and kinking, respectively

The dominant failure mechanism of penetration versus kinking is dictated by the lower value of fracture strength associated with each mode, recall (9) and (10). Upon equating ${\sigma ^{\infty }_{\mathrm{f}}}$ for the two modes, a failure mechanism map can be constructed, with axes $\sigma _{\mathrm{Y}}/\tau _{\mathrm{Y}}$ and $\mathrm {\Gamma }_{\mathrm{P}}/\mathrm {\Gamma }_{\mathrm{K}}$, for selected values of $a \sigma _{\mathrm{Y}}^{2}/E \mathrm {\Gamma }_{\mathrm{P}}$ and an assumed value of orthotropy $(\lambda , \rho )$. One such map for the case of isotropic solid is plotted in Fig. 10a for the crack and in Fig. 10b for the hole (again with $a/W=0.05$ and $H/W=1$). In each map, kinking is active when $\sigma _\mathrm{Y}/\tau _{\mathrm{Y}}$ and $\mathrm {\Gamma }_{\mathrm{P}}/\mathrm {\Gamma }_\mathrm{K}$ are large: kinking is encouraged by a low kinking strength $\tau _{\mathrm{Y}}$ and a low kinking toughness $\mathrm {\Gamma }_{\mathrm{K}}$ in relation to the values for penetration along the tensile cohesive zone. Mechanism maps similar to Fig. 10a can be constructed for each combination of $(\lambda , \rho )$ of the solid. We shall now show that some simplification is possible, such that the boundary between penetration and kinking can be collapsed onto approximately a single curve for each value of $a \sigma _{\mathrm{Y}}^{2}/E \mathrm {\Gamma }_{\mathrm{P}}$, upon suitably rescaling the toughness axis $\mathrm {\Gamma }_{\mathrm{P}}/\mathrm {\Gamma }_{\mathrm{K}}$ by a function $g_{1}(\lambda , \rho )$ and upon rescaling the strength axis $\sigma _{\mathrm{Y}}/\tau _{\mathrm{Y}}$ by a function $g_{2}(\lambda , \rho )$. The procedure for obtaining suitable scaling functions $g_{1}(\lambda , \rho )$ for the strength axis and $g_{2}(\lambda , \rho )$ for the toughness axis of the mechanism maps of the type shown in Fig. 10a, b is now described.

*Scaling for the toughness axis*: For plane elasticity problems, Suo et al. (1991) showed that the solutions for orthotropic elastic solids can be constructed from known isotropic solutions using a spatial rescaling technique. The purely energetic criterion of He and Hutchinson (1989) for crack deflection in an isotropic solid (recall (1)) was generalized to the orthotropic case such that the critical interface toughness ratio for crack penetration versus kinking in an orthotropic solid of Fig. 1a is given by

16 $$\begin{aligned} \dfrac{\mathrm {\Gamma }_{\mathrm{P}}}{\mathrm {\Gamma }_{\mathrm{K}}} = \dfrac{\lambda ^{\tfrac{1}{4}}}{G(\rho )} \end{aligned}$$

where $G(\rho )$ is obtained from an integral equation method as described in Wang et al. (1991). A regression analysis for the values of $G(\rho )$ listed in Wang et al. (1991) for $0.1 \le \rho \le 10$ gives the following functional form for $G(\rho )$:

17 $$\begin{aligned} {G(\rho )} = {(1+\rho )^{0.6}}+2.4 \end{aligned}$$

Accordingly, we rescale the toughness axis from ${\mathrm {\Gamma }_{\mathrm{P}}}/{\mathrm {\Gamma }_{\mathrm{K}}}$ to ${g_{1}(\lambda ,\rho )\mathrm {\Gamma }_{\mathrm{P}}}/{\mathrm {\Gamma }_\mathrm{K}}$ where

18 $$\begin{aligned} g_{1}(\lambda ,\rho ) = {\lambda ^{-\tfrac{1}{4}}}{G(\rho )} \end{aligned}$$

*Scaling for the strength axis*: Recall from Sect. 5.1 that there exists a lower bound value of strength ratio, $\sigma _{\mathrm{Y}}/\tau _{\mathrm{L}}$, below which plasticity within the shear band does not activate. The value of $\sigma _{\mathrm{Y}}/\tau _{\mathrm{L}}$ depends on $(\lambda , \rho )$ and a regression analysis of the calculations of Tankasala et al. (2017) for the semi-infinite crack show that for $(\lambda , \rho )$ such that $1 \le \rho \le 10$ and $1 \le \lambda \le 20$,

19 $$\begin{aligned} \dfrac{\sigma _{\mathrm{Y}}}{\tau _{\mathrm{YL}}} = \dfrac{1}{g_{2}(\lambda ,\rho )} = {\lambda ^{\tfrac{1}{4}}} H({\rho }) \end{aligned}$$

with

20 $$\begin{aligned} {H(\rho )} = {(1+\rho )^{0.5}}+1.8 \end{aligned}$$

Following the rescaling procedure as described above, we plot in Fig. 10c, d the mechanism maps for a sharp crack and a circular hole, respectively, using the rescaled abscissa $g_{2}(\lambda ,\rho )$ ${\sigma _{\mathrm{Y}}}/{\tau _{\mathrm{Y}}}$ and rescaled ordinate $g_{1}(\lambda ,\rho )$ ${\mathrm {\Gamma }_{\mathrm{P}}}/{\mathrm {\Gamma }_{\mathrm{K}}}$ . The solid black lines correspond to the isotropic case $(\lambda , \rho )=(1,1)$ while the solid gray lines correspond to the most extreme case of orthotropy considered in this study i.e., $(\lambda , \rho )=(20,5)$. The intermediate case of $(\lambda , \rho )=(1,10)$ lies within the gray shaded region. A number of broad remarks can be made by considering the maps of Fig. 10:

(i) The rescaling functions (18) and (20) for $g_{1}$ and $g_{2}$, respectively, yield almost a single boundary for the case of sharp crack with $a \sigma _{\mathrm{Y}}^{2}/E \mathrm {\Gamma }_{\mathrm{P}} \le 100$, see Fig. 10c. With decreasing crack length, the error between the rescaled boundaries increases for the two extreme values of $(\lambda , \rho )$.
(ii) For the case of a circular hole, the rescaling functions $g_{1}$ and $g_{2}$ do not coalesce the boundaries between penetration and kinking, except for the regime of $a \sigma _\mathrm{Y}^{2}/E \mathrm {\Gamma }_{\mathrm{P}} \le 0.1$ and $g_{2}(\lambda ,\rho )$ ${\sigma _{\mathrm{Y}}}/{\tau _{\mathrm{Y}}}$ $\ge 2$, as seen from Fig. 10d.
(iii) When $\sigma _{\mathrm{Y}}/\tau _{\mathrm{Y}}$ is sufficiently small, such as $g_{2}(\lambda , \rho )\sigma _{\mathrm{Y}}/\tau _{\mathrm{Y}} \le 1$, the kink band (i.e., the shear plastic zone) vanishes for any $\mathrm {\Gamma }_{\mathrm{P}}/\mathrm {\Gamma }_{\mathrm{K}}$, and only penetration can occur in a cracked solid. Penetration can also occur in the presence of shear bands provided $g_{1}(\lambda , \rho )\mathrm {\Gamma }_{\mathrm{P}}/\mathrm {\Gamma }_{\mathrm{K}}$ is sufficiently small, and for any value of $g_{2}(\lambda , \rho )\sigma _{\mathrm{Y}}/\tau _{\mathrm{Y}}>1$.
(iv) The threshold value of $g_{2}(\lambda , \rho )\sigma _\mathrm{Y}/\tau _{\mathrm{Y}}$, for which kinking cannot occur, is between 1.4 and 1.7 for the circular hole, thereby enlarging the field of dominance of penetration for the hole compared to that of the crack. Otherwise, there is only a relatively small effect of notch geometry upon the failure mechanism map for all assumed levels of orthotropy.
(v) Penetration is encouraged by a greater level of orthotropy for both the hole and the crack. This behaviour is consistent with the previous findings of Tankasala et al. (2017) for a semi-infinite crack.

## Case studies

There is a growing literature on the competition between cracking modes in both synthetic composites, and natural composites such as woods. We proceed to compare the predictions of the current study with the observations from the literature, first, for ceramic composites, and then, for natural hardwoods.

**Table 1:** Measured data for the ceramic fibre composites plotted in Fig. 10c

Label	Material	Source	$\lambda$	$\rho$	$\dfrac{\sigma _{\mathrm{Y}}}{\tau _{\mathrm{Y}}}$	$\dfrac{\mathrm {\Gamma }_{\mathrm{P}}}{\mathrm {\Gamma }_{\mathrm{K}}}$	Observed mode	Predicted mode
[1]	Monolithic SiC	Gogotsi (2003)	1	1	2	1	Penetration	Penetration
[2]	C–C	Evans and Zok (1994)	1	1	5	10	Kinking	Penetration or Kinking
[3]	SiC–SiC	Fang and Chou (1993)	1	1	5	300	Kinking	Kinking
[4]	Alumina–AS	Zhang et al. (2013)	1	3	1	15	Penetration	Penetration
[5]	Alumina–Mullite alumina	Mattoni and Zok (2005)	1	5.5	10	100	Kinking	Kinking
[6]	CFRP (UD layup)	Tan et al. (2015)	20	5	20	500	Kinking	Kinking

The table includes the labels used in Fig. 10c and gives the predicted and observed failure modes

**Table 2:** Scaling laws for the effective properties of wood, from Gibson and Ashby (1997)

Property	Scaling law	Value of the pre-exponent
Young’s modulus (in radial direction)	${E_{1}} = \alpha _{1} {\overline{\rho }}^{3}{E_{\mathrm{S}}}$	$\alpha _{1}=$ 0.8
Young’s modulus (in axial direction)	${E_{2}} = \alpha _{2}{\overline{\rho }}{E_{\mathrm{S}}}$	$\alpha _{2}=$ 1
Shear modulus (in axial-radial direction)	${G_{12}} = \alpha _{3} {\overline{\rho }}{E_{\mathrm{S}}}$	$\alpha _{3}=$ 0.074
Tensile strength (in axial direction)	${\sigma _{\mathrm{Y}}} = \alpha _{4} {\overline{\rho }}{\sigma _{\mathrm{YS}}}$	$\alpha _{4}=$ 0.34
Shear strength (in axial-radial direction)	${\tau _{\mathrm{Y}}} = \alpha _{5} {\overline{\rho }}{\sigma _{\mathrm{YS}}}$	$\alpha _{5}=$ 0.086
Fracture toughness normal to the grain (penetration), in MPa${\sqrt{\mathsf{m}}}$	$K_{\mathrm{IC}}^\mathrm{P} = \beta _{1} {\overline{\rho }}^{\tfrac{3}{2}}$	$\beta _{1}=$ 20 MPa${\sqrt{\mathrm{m}}}$
Fracture toughness along the grain (kinking), in MPa$\sqrt{{\mathsf {m}}}$	$K_{\mathrm{IC}}^\mathrm{K} = \beta _{2} {\overline{\rho }}^{\tfrac{3}{2}}$	$\beta _{2}=$ 1.8 MPa${\sqrt{\mathrm{m}}}$

### Ceramic composites

Ceramic–ceramic, long fibre composites such as SiC–SiC and oxide–oxide composites are beginning to enjoy use in the hot section of aerospace gas turbine engines due to their high temperature capability and low density compared to nickel-based superalloys. The high toughness of these materials is achieved through microstructural design such that crack kinking along the fibre direction occurs instead of penetration of the fibres.^2^ This is achieved in one of the following ways: (i) coating the fibres to form a weak interface between the matrix and the fibres (such as boron nitride coatings in SiC–SiC), (ii) increasing the level of matrix porosity (in Alumina-Aluminosilicate (AS) for example), and (iii) using fugitive coatings that volatalize during oxidation and leave a narrow gap at the fibre-matrix boundary, see Zok (2006) for a review of the developments in oxide–oxide composites. All of the above approaches result in a high value of penetration toughness $\mathrm {\Gamma }_{\mathrm{P}}$ due to fibre pull-out and a low value of kinking toughness $\mathrm {\Gamma }_{\mathrm{K}}$ due to a weak interface between fibre and matrix.

Consider, for example, the case of a plain weave of SiC fibres in a SiC matrix. This composite is approximately isotropic in in-plane elastic properties. Typically, the strength and toughness ratios relevant to the present study are $\sigma _{\mathrm{Y}}/\tau _{\mathrm{Y}}=5$ and $\mathrm {\Gamma }_{\mathrm{P}}/\mathrm {\Gamma }_{\mathrm{K}}=300$, see for example Droillard and Lamon (1996). Now place this data-point in Fig. 10c (and label it as [3]): kinking is predicted, in agreement with the observations of Fang and Chou (1993). Evans and Zok (1994) also observed crack kinking for a notched C–C composite with isotropic in-plane elastic properties but $\sigma _{\mathrm{Y}}/\tau _\mathrm{Y}=5$ and $\mathrm {\Gamma }_{\mathrm{P}}/\mathrm {\Gamma }_{\mathrm{K}}\approx 10$. We find that this data-point, labelled [2], lies on the boundary between penetration and kinking, indicating equal likelihood of failure by penetration or kinking. In contrast, a monolithic and isotropic SiC ceramic typically has the properties $\sigma _{\mathrm{Y}}/\tau _{\mathrm{Y}}=2$ and $\mathrm {\Gamma }_\mathrm{P}/\mathrm {\Gamma }_{\mathrm{K}}\approx 1$ (label [1] in Fig. 10c) and crack penetration is predicted, in agreement with common experience (see for example, Gogotsi 2003).

Second, consider a composite such that $(\lambda ,\rho )=(1,5.5)$, for example an oxide–oxide composite of alumina fibres in a mullite/alumina matrix. As for SiC–SiC, this composite exists as a plain orthogonal weave, but now the matrix is designed to possess a low modulus and low toughness by the introduction of a high level of porosity (about 30%). Consequently, $\sigma _{\mathrm{Y}}/\tau _\mathrm{Y}=10$ and $\mathrm {\Gamma }_{\mathrm{P}}/\mathrm {\Gamma }_{\mathrm{K}}=100$, as estimated from the data of Mattoni and Zok (2005). Again, this combination of material parameters gives rise to crack kinking, see label [5] in Fig. 10c. This is in contrast to an alumina-aluminosilicate composite for which $\sigma _\mathrm{Y}/\tau _{\mathrm{Y}}=1$ so that crack growth occurs by penetration for $a\sigma ^{2}_{\mathrm{Y}}/E\mathrm {\Gamma }_{\mathrm{P}}=0.2$, as observed by Zhang et al. (2013); this is labelled as [4] in Fig. 10c.

Third, consider the highly anisotropic case of a unidirectional carbon fibre in epoxy lamina such that $\lambda =20$ and $\rho =5$. A high penetration toughness $\mathrm {\Gamma }_{\mathrm{P}}$ is associated with extensive fibre pull-out. Crack deflection is predicted correctly in Fig. 10c (see label [6]) upon making use of the representative values $\sigma _{\mathrm{Y}}/\tau _{\mathrm{Y}}=20$ and $\mathrm {\Gamma }_{\mathrm{P}}/\mathrm {\Gamma }_{\mathrm{K}}=500$ (as taken from Min-Seok and Xiao-Zhi 1994; Tan et al. 2016). The various cases of composites considered here are summarized in Table 1, along with the predicted and observed modes of failure.

### Natural hardwoods

Natural woods are cellular solids with hexagonal prismatic cell walls with the relative density ${\overline{\rho }}$ (density of the wood divided by that of the cell wall material) ranging from 0.13 for balsa to 0.84 for lignum vitae. At a micron scale, each cell wall is a fibre-reinforced composite with crystalline cellulose fibres embedded in an amorphous hemicellulose and lignin matrix. The layup of the fibres in the cell walls, along with the anisotropic shape of the cells, give rise to extreme anisotropy in the mechanical properties of wood. Most natural woods have three orthogonal planes of symmetry: axial (parallel to the trunk), radial, and tangential. The stiffness and strength are largest in the axial direction and smallest in the tangential direction, in a ratio of up to 20 depending upon the species of wood (Desch and Dinwoodie 1981). Additionally, the fracture toughness of wood is direction-dependent: it is up to 10 times larger for cracks that propagate across the grain than for cracks that kink along the grain, see Ashby et al. (1985). This anisotropy in elastic properties and in toughness make natural woods an ideal orthotropic solid for comparison with the predictions of the current study.

**Table 3:** Orthotropy constants $(\lambda$,$\rho )$ and toughness ratio ${\mathrm {\Gamma }_{\mathrm{P}}}/{\mathrm {\Gamma }_{\mathrm{K}}}$ for natural hardwoods based on the scaling laws provided in Table 2

Species of hardwood	Density (kg/m$^{3}$)	Relative density, ${\overline{\rho }}$	$\lambda$	$\rho$	${\mathrm {\Gamma }_{\mathrm{P}}}/{\mathrm {\Gamma }_{\mathrm{K}}}$
Balsa	200	0.13	70.3	1.6	1.7
Aspen	300	0.2	31.2	2.4	3.9
Yellow poplar	380	0.25	19.5	3.1	6.3
Khaya	440	0.3	14.5	3.5	8.5
Oak	580	0.38	8.3	4.7	14.8
Birch	620	0.41	7.3	5	16.9
Ash	670	0.44	6.2	5.4	19.7
Beech	750	0.5	5	6	24.7

The data are inferred from Gibson and Ashby (1997)

In this section, we develop case studies for various natural hardwoods containing a finite pre-crack to assess the competition between penetration (across the grain) and kinking (along the grain). Assume that a pre-crack is aligned with the radial direction of the wood $x_{1}$ such that kinking may occur along the axial direction $x_{2}$. The relevant effective mechanical properties for wood are estimated from the scaling laws as proposed by Gibson and Ashby (1997); these are tabulated in Table 2 in terms of the relative density of the wood ${\overline{\rho }}$, axial cell-wall Young’s modulus $E_{\mathrm{S}}$, and cell-wall yield strength $\sigma _{\mathrm{YS}}$.

In order to deploy the failure mechanism map of Fig. 10c, we need (i) the orthotropic constants $(\lambda , \rho )$, (ii) the strength ratio ${\sigma _{\mathrm{Y}}}/{\tau _{\mathrm{Y}}}$, and (iii) the toughness ratio ${\mathrm {\Gamma }_{\mathrm{P}}}/{\mathrm {\Gamma }_{\mathrm{K}}}$ for each choice. The orthotropic constants $(\lambda , \rho )$ follow from (8) as

21 $$\begin{aligned} \lambda \approx \dfrac{E_{2}}{E_{1}} = \dfrac{\alpha _{2}}{\alpha _{1}} \dfrac{1}{{\overline{\rho }}^{2}} \end{aligned}$$

and

22 $$\begin{aligned} \rho \approx \dfrac{\sqrt{E_{1}E_{2}}}{G_{12}} = \dfrac{\sqrt{\alpha _{1}\alpha _{2}}}{\alpha _{3}} {\overline{\rho }} \end{aligned}$$

where the pre-exponents $(\alpha _{1},\alpha _{2}, \alpha _{3})$ are given in Table 2. It is seen from the expressions for tensile strength $\sigma _{\mathrm{Y}}$ and the shear strength $\tau _\mathrm{Y}$ in Table 2 that the strength ratio ${\sigma _{\mathrm{Y}}}/{\tau _{\mathrm{Y}}}$ is constant for all values of $(\lambda , \rho )$ such that

23 $$\begin{aligned} \dfrac{\sigma _{\mathrm{Y}}}{\tau _{\mathrm{Y}}} = \dfrac{\alpha _{4}}{\alpha _{5}} = 3.95 \end{aligned}$$

The penetration toughness $\mathrm {\Gamma }_{\mathrm{P}}$ is the mode I toughness for a crack oriented along the radial direction $x_{1}$; this is given in terms of the corresponding fracture toughness $K_{\mathrm{IC,P}}$ and the orthotropic constants $(\lambda , \rho )$ according to Suo et al. (1991) as

24 $$\begin{aligned} \mathrm {\Gamma }_{\mathrm{P}} = \lambda ^{\tfrac{1}{4}} \sqrt{\dfrac{(1+\rho )}{2}} \dfrac{{K_{\mathrm{IC}}^\mathrm{P}}^{2}}{E_{2}} = \lambda ^{\tfrac{1}{4}} \sqrt{\dfrac{(1+\rho )}{2}} \dfrac{\beta _{1}^{2}}{\alpha _{2}} \dfrac{{\overline{\rho }}^{2}}{E_\mathrm{S}} \nonumber \\ \end{aligned}$$

where the pre-exponent $\beta _{1}$ is specified in Table 2. As for the kinking toughness $\mathrm {\Gamma }_{\mathrm{K}}$, we assume that $\mathrm {\Gamma }_{\mathrm{K}}$ is adequately given by the mode I toughness for a crack oriented along the axial direction $x_{2}$. Accordingly, the relationship between the corresponding fracture toughness $K_{\mathrm{IC,K}}$, and $(\lambda , \rho )$ as taken from Suo et al. (1991) has the form

25 $$\begin{aligned} \mathrm {\Gamma }_{\mathrm{K}} = \lambda ^{\tfrac{5}{4}} \sqrt{\dfrac{(1+\rho )}{2}} \dfrac{{K_{\mathrm{IC}}^\mathrm{K}}^{2}}{E_{2}} = \lambda ^{\tfrac{5}{4}} \sqrt{\dfrac{(1+\rho )}{2}} \dfrac{\beta _{2}^{2}}{\alpha _{2}} \dfrac{{\overline{\rho }}^{2}}{E_\mathrm{S}} \nonumber \\ \end{aligned}$$

where the value of $\beta _{2}$ is given in Table 2.

The interface toughness ratio ${\mathrm {\Gamma }_{\mathrm{P}}}/{\mathrm {\Gamma }_{\mathrm{K}}}$ then follows from (24) and (25) as

26 $$\begin{aligned} \dfrac{\mathrm {\Gamma }_{\mathrm{P}}}{\mathrm {\Gamma }_{\mathrm{K}}} = \left( \dfrac{\beta _{1}}{\beta _{2}} \right) ^{2} \dfrac{1}{\lambda } \end{aligned}$$

Based on the expressions listed above, we tabulate in Table 3 the values of $(\lambda ,\rho )$, and ${\mathrm {\Gamma }_{\mathrm{P}}}/{\mathrm {\Gamma }_{\mathrm{K}}}$ for various natural hardwoods using data from Ashby et al. (1985). In doing so, we assume a cell-wall density of 1500 kg/m$^{3}$, axial cell-wall Young’s modulus, $E_{\mathrm{S}}= 35$ GPa and cell-wall yield strength, $\sigma _{\mathrm{YS}}= 350$ MPa. The strength ratio in all cases is ${\sigma _{\mathrm{Y}}}/{\tau _{\mathrm{Y}}}$ $=3.95$, recall (23). Upon placing the data for this selection of woods on the map of Fig. 10c, we find that the dominant mode of failure is always penetration across the grain. This prediction is consistent with observations on notched specimens of wood wherein the initial crack growth occurs by penetration, see for example, Fig. 10.23(b) of Gibson and Ashby (1997).

## Concluding remarks

Many synthetic and natural composites are orthotropic and contain interfaces of low strength and low toughness such that damage zones at the notch tip are on the order of the notch size (Heredia et al. 1994; Tan et al. 2015). Thus, the use of a macroscopic toughness alone to design a notched composite is of limited use. Further, a large class of engineering solids fail by the *simultaneous* development of one or more inelastic damage zones at the crack tip. In the present study, we have investigated the macroscopic tensile strength of a panel with a centre crack or circular hole associated with the simultaneous activation of tensile and shear damage zones. The simple approach adopted herein of two simultaneous damage zones is able to account for several experimentally observed phenomena. These include: (i) the hole size effect, (ii) sensitivity to notch acuity, and (iii) the role of elastic orthotropy. The role of the relative penetration to kinking strength and relative toughness in promoting failure via extension by penetration versus splitting is quantified in failure mechanism maps for a sharp crack and a circular hole.

The main findings of the present study are as follows.

- Crack penetration is promoted over kinking for sufficiently small defects (crack or hole), for the full range of orthotropy studied.
- With increasing orthotropy, the penetration mode is promoted for any given flaw size (crack or hole).
- Commonly, the strength ratio $\sigma _{\mathrm{Y}}/\tau _{\mathrm{Y}}$ is large for orthotropic solids and this leads to large shear bands relative to the tensile plastic zones, $\ell _{\mathrm{K}}^\mathrm{f}/\ell _{\mathrm{P}}^\mathrm{f} \gg 1$. The long shear bands shield the crack tip and elevate the tensile strength for failure by penetration. In contrast, the length of the tensile plastic zone has only a minor effect on the failure strength due to kinking: ${\sigma ^{\infty }_{\mathrm{f}}}/\tau _{\mathrm{Y}}$ is a function of $a \tau _{\mathrm{Y}}^{2}/E \mathrm {\Gamma }_{\mathrm{K}}$ with only a minor sensitivity to the magnitude of $\sigma _{\mathrm{Y}}/\tau _{\mathrm{Y}}$.
- The cohesive zone model gives insight into the observed failure modes of notched fibre composites and natural hardwoods. However, we emphasize that the theory holds for a much broader range of orthotropic solids such as drawn polymers and anisotropic ceramic crystals.

We also emphasize that the scope of the present study is limited to the case when the kink band deforms and fails in shear. The nature of the cohesive zone will depend upon the micromechanism of failure in any material system. The competition between kinking and penetration will be affected by the choice of the cohesive zone law for the kink bands, and this could be explored in a future study.

## Footnotes

1 In the context of fibre-reinforced composite materials, this configuration corresponds to a notch perpendicular to the main $0^{\circ }$ fibres.

2 In fact, some crack advance may occur directly ahead of the parent crack but the high pull-out toughness gives a high ratio of $\mathrm {\Gamma }_{\mathrm{P}}/\mathrm {\Gamma }_{\mathrm{K}}$, thereby encouraging crack kinking.
